# Organophosphine-Catalyzed [4C+X] Annulations

**DOI:** 10.3390/molecules23113022

**Published:** 2018-11-19

**Authors:** Yangyan Li, Xiang Chen, Xiaoming Chen, Xiao Shen

**Affiliations:** 1 College of Chemistry and Bioengineering, Hunan University of Science and Engineering, Yongzhou 425199, Hunan, China; liyangyan2@163.com (Y.L.); chenxiaoming3012@163.com (X.C.); 2 Institute for Advanced Studies, Wuhan University, 299 Bayi Road, Wuchang District, Wuhan 430072, Hubei, China; lucasphe@163.com

**Keywords:** phosphine catalysis, annulation, *C*_4_ synthons

## Abstract

In recent years, there have been extraordinary developments of organophosphine-catalyzed reactions. This includes progress in the area of [4C+X] annulations, which are of particular interest due to their potential for the rapid construction of 5–8-membered cyclic products. In this short overview, we summarize the remarkable progress, emphasizing reaction mechanisms and key intermediates involved in the processes. The discussion is classified according to the type of electrophilic reactants that acted as *C*_4_ synthons in the annulation process, in the order of α-alkyl allenoates, γ-alkyl allenoates, α-methyl allene ketones, β′-OAc allenoate, δ-OAc allenoate, activated dienes and cyclobutenones.

## 1. Introduction

The continuous innovation of synthetic methodologies is one of the sources for sustainable progress in modern organic chemistry. Since the Diels–Alder reaction was reported in 1928, cycloaddition reactions have evolved into one of the most fascinating fields in organic chemistry because of their powerful applications in convergent synthesis of cyclic compounds from simple starting materials. Apart from the electrocyclic reactions, nucleophilic phosphine catalysis, which go through different dipolar cycloaddition modes, also played a significant role in the development of cycloaddition chemistry [1,2,3,4,5,6,7,8,9,10,11,12,13,14,15,16,17,18,19,20,21,22]. Those novel annulations provide efficient and powerful approaches to a wide array of carbo- and heterocyclic motifs which are frequently found in natural products as well as biologically active molecules, usually in a regio- and stereoselective manner [1,2,3,4,5,6,7,8,9,10,11,12,13,14,15,16,17,18,19,20,21,22]. In addition, due to their merits such as readily availability of starting materials, mild and metal-free reaction conditions, simple post-reaction workup, high atom economy, low environmental pollution, etc., the tertiary phosphine-promoted cycloaddition reactions have received extensive interest from synthetic chemists, and become an important means in sustainable organic synthesis.

In 1995, Lu et al. first reported the PPh_3_-catalyzed [3+2] cycloaddition reactions of 2,3-butadienoates or 2-butynoates with electron-deficient olefins for the preparation of cyclopentenes [23]. Intrigued by this pioneering work, the potential of allenes acting as a type of *C*_3_ synthons were further exploited. Since then, a wide variety of [3+2] annulations together with their asymmetric catalytic versions have been developed. Further studies disclosed that allenes could also play the roles of *C*_2_ [24,25,26,27,28,29,30,31,32,33] and *C*_1_ [34,35,36] units in various cycloadditions under some circumstances. In addition to allenes, electron-deficient alkynes, and MBHADs (Morita–Baylis–Hillman alcohol derivatives) were often used as *C*_3_ synthons in the tertiary phosphine catalyzed [3+2] cycloaddition reactions with electron-poor olefins or imines as well. With the help of newly developed chiral phosphine catalysts, many synthetic methodologies have been developed for the construction of optically active five-membered ring systems [21].

Thanks to the readily available *C*_3_ synthons (e.g., allenes, electron-deficient alkynes, and MBHADs), phosphine catalyzed [3C+X] cycloadditions have been extensively studied. However, the development of organophosphine-catalyzed [4C+X] cycloadditions is lagging far behind. One of the reasons might be the lack of suitable *C*_4_ synthon. Since Kwon and coworkers reported the innovative finding about the employment of α-substituted allenoates as a novel type of *C*_4_ synthon [37], tremendous progress has been made in the field of organophosphine-catalyzed [4C+X] annulations. Those annulation reactions provide efficient way for the rapid construction of 5–8-membered cyclic products, which are of particular importance in organic synthesis. However, there has been no specific review on this topic. Herein, we present a comprehensive review to summarize the organophosphine-catalyzed [4C+X] annulations as well as to elaborate the mechanisms and key intermediates involved in the process. This overview covers all kinds of nucleophilic phosphine-catalyzed [4C+X] annulations, including [4+1], [4+2], [4+3], and [4+4] cycloadditions and some other miscellaneous [4C+X] annulations. The discussion is organized according to the type of electrophilic reactants that acted as *C*_4_ synthons in the annulation process, in the order of α-alkyl allenoates, δ-aryl allenoates, α-methyl allene ketones, β′-OAc allenoate, δ-OAc allenoate, activated 1,3-dienes and cyclobutenones.

## 2. [4+X] Annulations of α-Alkyl Allenoates (or 2-Alkyl 2,3-Butadienoates)

**Scheme 1:**
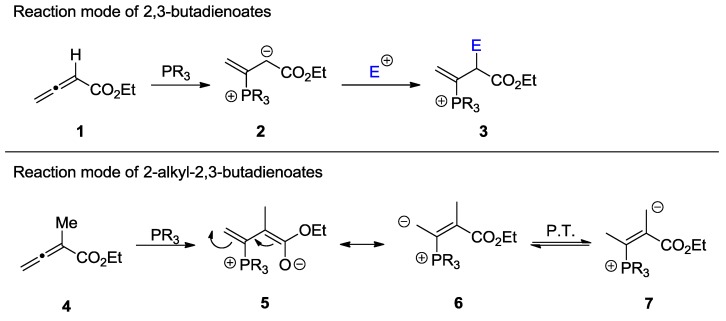
Different reaction mode between 2,3-butadienoates and 2-substituded 2,3-butadienoates.

Ordinarily, organophosphine-catalyzed reactions of 2,3-butadienoates are initiated by the α-addition of the zwitterionic intermediate **2** to other electrophiles. However, substitution of the hydrogen at the C2 position of 2,3-butadienoates with a methyl group blocked the α-attack of the zwitterionic intermediate. Instead, unprecedented reaction modes initiated by γ- or β′-addition of the zwitterionic intermediate to other electrophiles usually occurred (Scheme 1). Thus, 2-alkyl-2,3-butadienoates act as a type of 1,4-dipole synthon in the annulation with activated imines, electron-deficient olefins, ketones and azomethine imines, rather than the traditional role of 1,3-dipolar synthon in the non-substituted allenoates.

### 2.1. [4+2] Annulations of α-Alkyl Allenoates with Activated Imines

**Scheme 2:**
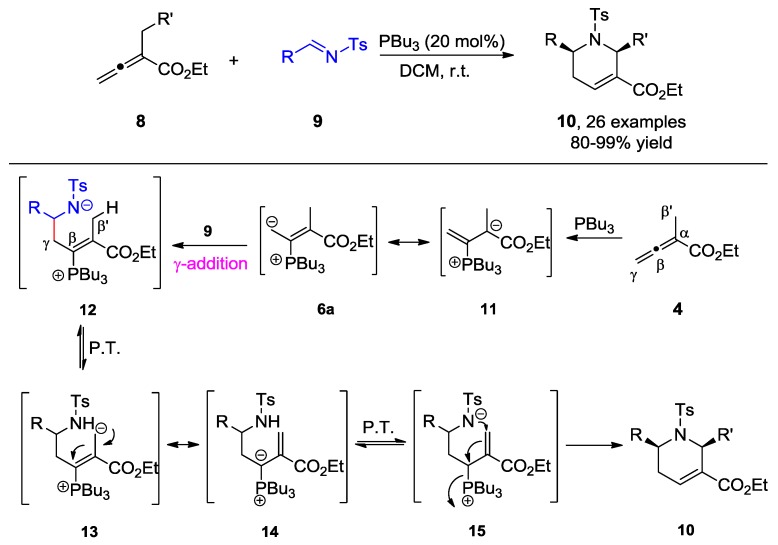
Proposed mechanism of the [4+2] annulation reaction [37].

The [4+2] annulations of α-alkyl allenoates with sulfonimides resulted in the production of highly functionalized tetrahydropyridines [37]. The mechanism of the [4+2] annulation reaction is outlined in Scheme 2: Firstly, nucleophilic addition of PBu_3_ to the β-position of α-alkyl allenoates resulted in the formation of zwitterionic intermediate **11**, which existed as a resonance-stabilized zwitterionic intermediate **6a**. Subsequently, the allylic carbanion **6a** underwent a nucleophilic addition to the imine **9** at the γ-position of the allenoate to produce intermediate **12**. It was supposed that the reason for the γ-position selectivity was that the larger steric hindrance of the α-position blocked its corresponding nucleophilic attack. Then, two consecutive proton-transfer processes caused the shift of C-C double bond. Finally, 6-*endo* ring closure of intermediate **15** accompanied with the release of phosphine catalyst generated the product. The 2-alkyl-2,3-butadienoates played the role of 1,4-dipole synthon throughout the whole reaction process. This important finding extended the scope of phosphine-catalyzed annulations of allenoates from [3C+X] to [4C+X] cycloadditions. It represents the milestone for the role-transformation of allenoates, and lays the foundation for the subsequent similar 1,4-dipolar cycloaddition reactions.

**Scheme 3:**
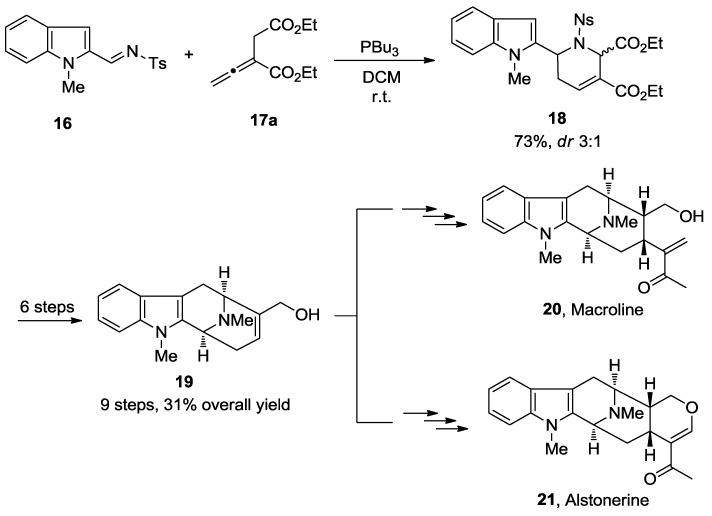
Formalsynthesis of alkaloids (±)-macroline and (±)-alstonerine [38,39,40,41].

**Scheme 4:**
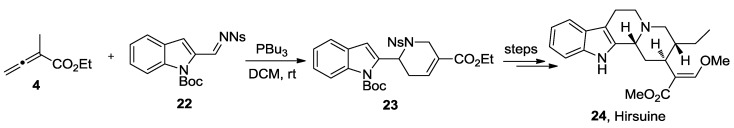
Total synthesis of alkaloids (±)-Hirsutine [42].

In 2005, the above phosphine-catalyzed [4+2] cycloaddition of α-alkyl allenoate was successfully applied to the formal synthesis of *Alstonia* macroline alkaloids, (±)-macroline and (±)-alstonerine by Kwon et. al. Under the catalysis of PBu_3_, [4+2] annulation of diester allene **17a** with indoly-substituted imine **16** gave rise to indoly-substituted tetrahydropyridine **18** in 73% yield with 3:1 *dr*, which was then converted to the key allyl alcohol compound **19** by a sequence of six-step transformations. Since compound **19** was the known key intermediate in the total synthetic route of (±)-macroline (**20**) and (±)-alstonerine (**21**) reported by the group of Cook [38,39,40], Kwon and coworkers’ research work represents formal synthesis of alkaloids (±)-macroline and (±)-alstonerine (Scheme 3) [41]. Besides, a similar [4+2] cycloaddition of ethyl α-methyl allenoate with imine was also applied to the total synthesis of (±)-Hirsutine by the same group in 2012 (Scheme 4) [42].

**Scheme 5:**
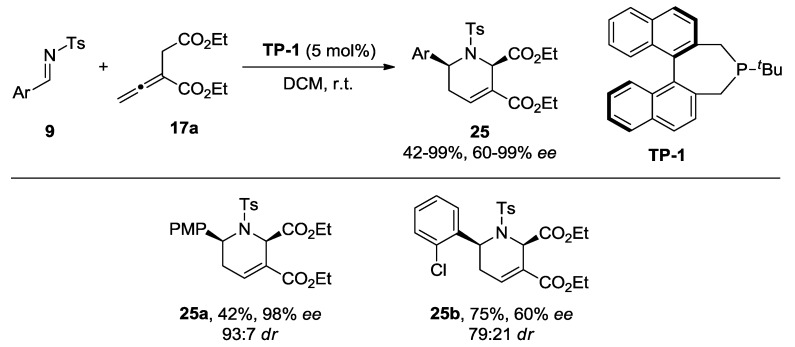
Catalytic asymmetric [4+2] annulation of α-alkyl allenoates with imines [43].

In 2005, the group of Fu achieved the asymmetric version of Kwon’s [4+2] annulation of α-alkyl allenoates with imines for the first time by using an axial chiral tertiary phosphine catalyst **TP**-**1** with a binaphthyl skeleton (Scheme 5) [43]. For most substrates, this reaction has excellent diastereoselectivity, moderate to excellent yields and *ee* values. However, the electron-rich *p*-methoxybenzaldehyde-derived imine appears to be relatively unreactive and can only achieve 42% yield of target product **25a**, although the *ee* value is as high as 98%. Besides, imines with electron-poor substituents on the *ortho*-position of the aromatic groups resulted in unsatisfactory stereoselectivities (e.g., **25b**). 

**Scheme 6:**
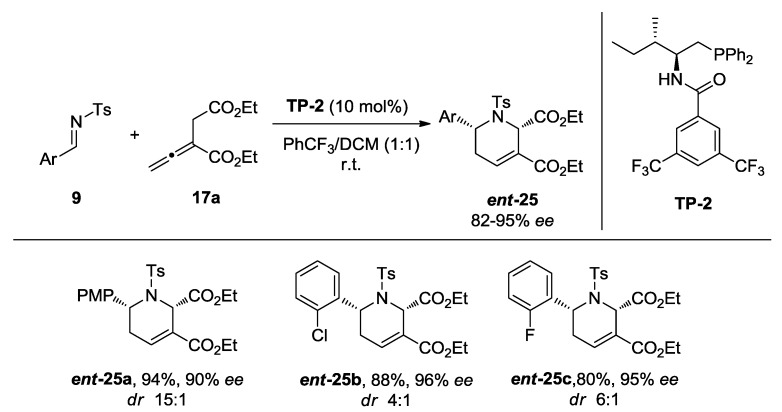
Catalytic asymmetric [4+2] annulation of α-alkyl allenoates with imines (PMP: *p*-methoxyphenyl) [44].

The asymmetric [4+2] cycloaddition of α-alkyl allenoates with *N*-tosyl aldimines were achieved by Zhao et. al. as well in 2011 (Scheme 6) [44]. A simple bifunctional *N*-acyl amino phosphine catalyst **TP**-**2** derived from isoleucine was identified to be the best catalyst for the [4+2] cycloaddition, providing a series of chiral tetrahydropyridines in good to high enantioselectivities whose absolute configuration was just opposite to those reported by the Fu’s group. It is worth noting that the electron-rich 4-anisyl imine **9a** which was once a reluctant coupling partner in Fu’s catalytic system worked as well and gave the corresponding product ***ent***-**25a** in an excellent yield and 90% *ee*. 2-chlorobenzene aldimine **9b**, which performed unsatisfactorily previously, also gave an improved result: from 75% to 88% yield and from 60% to 96% *ee*. 

**Scheme 7:**
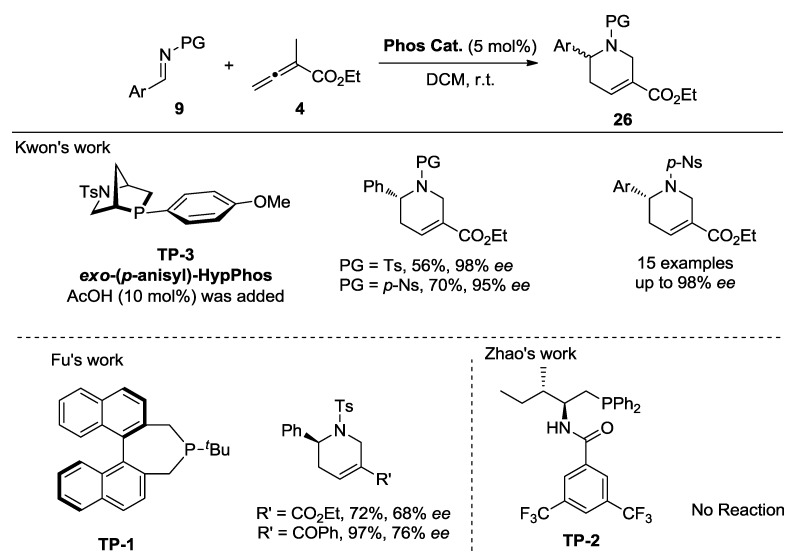
Catalytic asymmetric [4+2] annulation of α-methyl allenoates with imines [43,44,46].

**Scheme 8:**
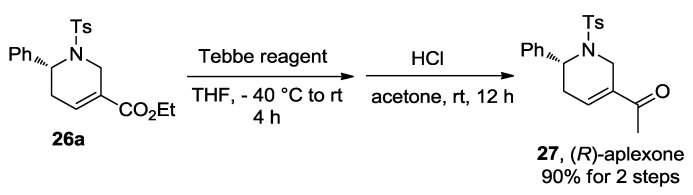
Asymmetric synthesis of (*R*)-aplexone [46].

In 2014, the group of Kwon developed a class of *trans*-hydroxy-*_L_*-proline (Hyp) derived P-chiral [2.2.1] bicyclic phosphine catalysts, which was named as “HypPhos” ligands [45]. Recently, they applied the HypPhos to the [4+2] annulation of α-alkyl allenoates with imines, and found that *exo*-(*p*-anisyl)-HypPhos **TP**-**3** was able to effect the above [4+2] annulation to produce tetrahydropyridines in excellent enantioselectivities (Scheme 7) [46]. Although the highly enantioselective synthesis of 2,6-disubstituted guvacines had been achieved by Fu’s and Zhao’s groups. optically active 6-substituted (none substitution at the *C*_2_ position) guvacines could not be acquired as easy as the former. In Fu’s research, 6-substituted guvacines **26** were formed in moderate enantioselectivities (Scheme 7) [43], and under the catalytic system of Zhao’s amino acid derived bifunctional phosphine **TP**-**2**, the 6-phenyl guvacine ester was not obtained [44]. However, Kwon and coworkers’ catalytic system overcame the limitation and achieved the [4+2] annulation of α-methyl allenoates **4** with imines to produce 6-substituted guvacine esters in excellent enantioselectivities [46]. With this method, optically enriched (*R*)-aplexone **27**, which is a promising anti-cholesterol drug, could be quickly synthesized through a two-step, high-yielding sequential transformation of the chiral 6-disubstituted guvacines **26a** (Scheme 8).

**Scheme 9:**
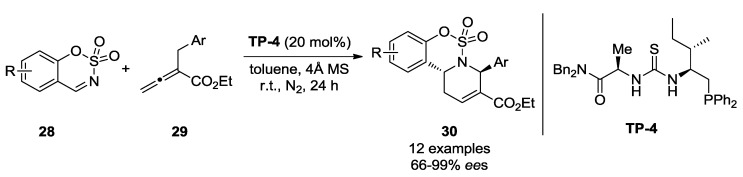
[4+2] cycloaddition of α-alkyl allenoates withsulfamate-derived cyclic imines [47].

In 2014, Guo’s group achieved a [4+2] cycloaddition reaction of sulfamate-derived cyclic imines with α-alkyl allenoates (Scheme 9) [47]. For the racemic version, *n*-PrPPh_2_ was proved as the best catalyst that effected the reaction to provide a variety of sulfamate-fused tetrahydropyridines **30** in high yield with excellent diastereoselectivities. Similar to previous mechanisms proposed by Kwon, Fu, Zhao, the zwitterionic intermediate exhibited normal regioselectivity: nucleophilic attack took place at the γ-position. When a chiral bifunctional tertiary phosphine catalyst **TP**-**4** derived from natural amino acids was employed as the catalyst, the asymmetric [4+2] cycloaddition proceeded smoothly to afford a series of chiral sulfamate-fused tetrahydropyridines **30** in good yields with moderate to excellent enantioselectivities.

**Scheme 10:**
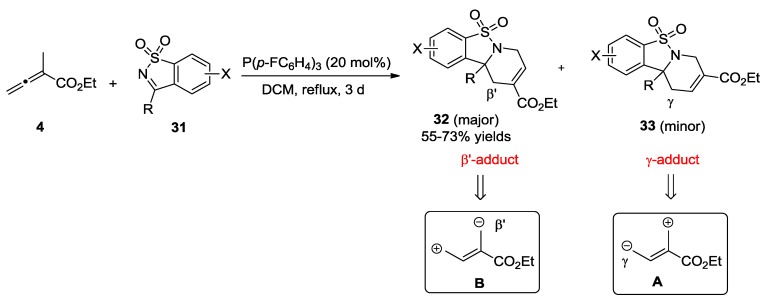
Thermodynamically favored [4+2] cycloaddition of α-alkyl allenoates with cyclic ketimines [48].

In 2012, a different [4+2] cycloaddition of 2-methyl-2,3-butadienoate **4** with cyclic saccharin-derived ketimines **31** was reported by the group of Ye (Scheme 10) [48]. Nucleophilic attack of the zwitterionic intermediate exhibited β′-position selectivity rather than the γ-selectivity that was favored in other [4+2] cycloaddition of α-methyl allenoate with imines. Thus, in the whole reaction process, α-alkyl allenoate was equal to type-**B** 1,4-dipole which posed a reversed polarity compared to previously introduced [4+2] annulations. The electronic property of the phosphine catalyst was conjectured to be the determining factor for the regioselectivity: electron-poor phosphine catalysts are inclined to promote β′-addition while electron-rich nucleophilic catalysts usually lead to γ-addition.

**Scheme 11:**
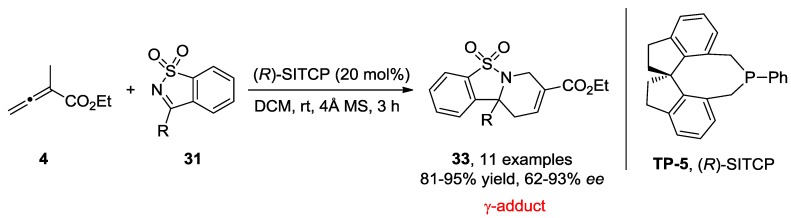
Kinetically favored [4+2] cycloaddition of α-alkyl allenoates with cyclic ketimines [49].

The enantioselective [4+2] cycloadditions of 2-methyl-2,3-butadienoate **4** with saccharin-derived ketimines **31** was also realized by Sasai and coworkers in 2014 (Scheme 11) [49]. The highly nucleophilic monoaryl phosphine catalyst (*R*)-SITCP (**TP**-**5**) developed by Zhou’s group catalyzed the enantioselective [4+2] cycloadditions in a normal γ-selective addition pathway, producing kinetically favored multifunctionalized tetrahydropyridines **33** with a hetero-quaternary chiral center in high yields with up to 93% *ee* and ≥20:1 regioselectivities. 

### 2.2. [4+2] Annulations of α-Alkyl Allenoates with Electron-Deficient Olefins

**Scheme 12:**
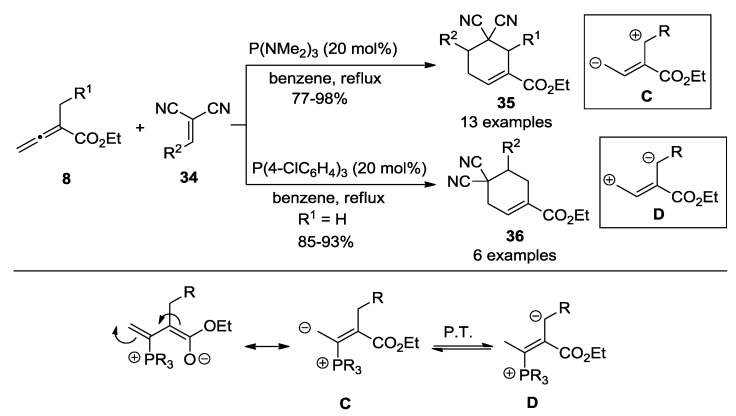
[4+2] cycloaddition of α-alkyl allenoates with arylidenemalononitrile [50].

Under the catalysis of organophosphines, α-alkyl allenoates, as effective novel *C*_4_ synthons, reacted not only with activated imines to produce tetrahydropyridines, but could also couple with electron-deficient alkenes to prepare all-carbon cyclohexenes. In 2007, Kwon et al. realized the organophosphine-catalyzed [4+2] cycloaddition of 2-alkyl allenoates with arylidenemalononitrile **34** (Scheme 12) [50]. Interestingly, a reversal of regioselectivity was found when different organophosphine catalysts were employed: the stronger nucleophilic hexamethylphosphine triamide (HMPT) effected the annulation reaction with normal γ-position selectivity while the less nucleophilic triarylphosphines with electron-deficient aryl groups converted the α-alkyl allenoates to an inverted 1,4-dipole equivalent **D** (β′-position selectivity). The explanation for this intriguing reversal of regioselectivity was that the phosphonium dienolate-to-phosphorus ylide equilibrium (**C** to **D**) favored the ylide **D** when a more electron-withdrawing triarylphosphine was utilized.

**Scheme 13:**
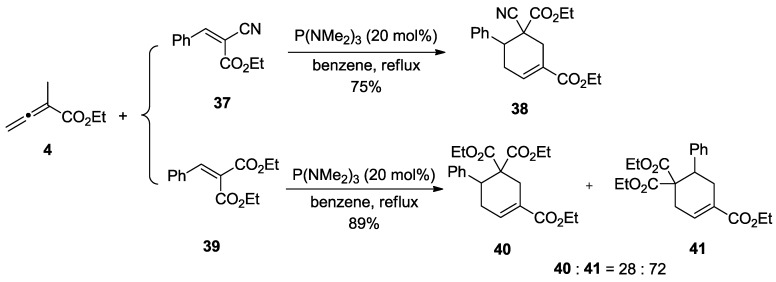
[4+2] annulations of 2-methyl allenoate with arylidene cyanoacetate or arylidene malonates [51].

Later, more extensive electrophilic olefins were used in the [4+2] annulation reaction by Kwon and coworkers (Scheme 13) [51]. Similarly, HMPT was identified as the best nucleophilic phosphine catalyst. Reaction of arylidene cyanoacetates **37** with 2-methyl allenoate **4** afforded the normal γ-position cycloadducts **38**. However, when the less-reactive arylidene malonates **39** were employed as the olefin substrates, both cycloadducts **40** and **41** resulted from two different reaction pathways were produced. It was rationalized that there was enough time for the isomerization of phosphonium dienolate **C** into vinylogous ylide **D** when relatively unreactive olefin substrates were used. It should be noted that triarylphosphine or some other electron-rich tertiary phosphines were ineffective at mediating the cycloaddition reactions.

In 2012, the group of Lu and Zhao independently realized the first asymmetric [4+2] annulation reaction of α-alkyl allenoates with activated olefins by employing the amino acid-derived LB-BA (short for Lewis base-Brønsted acid) bifunctional chiral tertiary phosphine catalyst.

**Scheme 14:**
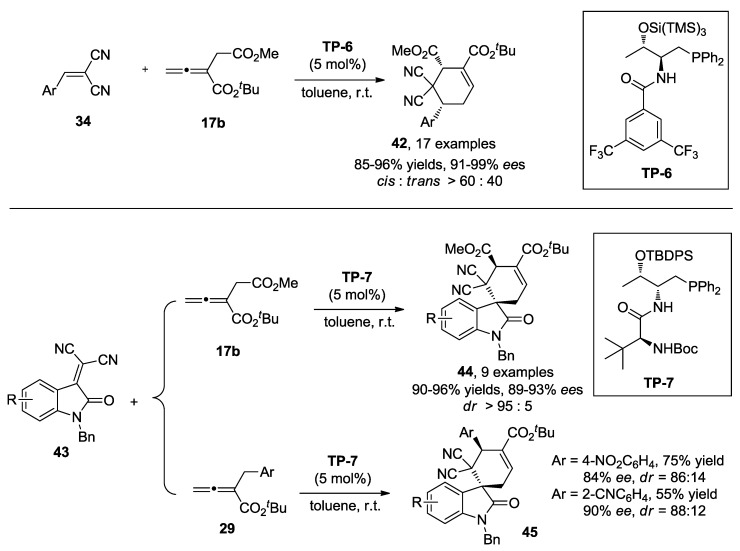
Asymmetric [4+2] cycloaddition of α-alkylallenoates with activated olefins [52].

In the research work of Lu (Scheme 14) [52], two different types of LB-BA bifunctional chiral phosphine catalysts were identified to be suitable for two different types of activated alkene substrates: for the [4+2] cycloaddition of arylidene malononitrile **34**, threonine-derived bifunctional chiral tertiary phosphine catalyst **TP**-**6** with a sterically bulky tris(trimethylsilyl)silyl (TTMSS) group on the oxygen atom was found to be the best catalyst, enabling the formation of cyclohexenes **42** in high to excellent yields with excellent enantioselectivities and moderate to good diastereoselectivities, while, for the [4+2] cycloaddition of isatylidenemalononitrile **43**, the performance of the chiral tertiary phosphine catalyst **TP**-**6** was not ideal as only 17% *ee* value could be induced, however, the chiral tertiary phosphine catalyst **TP**-**7** based on the skeleton of dipeptide *O*-TBDPS-*_D_*-Thr-*_L_*-*N*-Boc-*tert*-Leu was adequate to mediate the annulation and provide the desired 3-spirocyclohexene-2-oxidoles **44** and **45** in high yield with excellent enantioselectivities and diastereoselectivities. Due to the ester group at the β′ position of the allenoates, which increases the steric hindrance of the β′ position, γ-addition cycloadducts were formed exclusively for the two types of [4+2] cycloadditions. After replacement of the ester group at the β′ position with electron-poor aromatic rings, the [4+2] annulation could also proceed smoothly to afford cyclization products in moderate yields with good *dr* and excellent *ee*. However, β′-substitution of electron neutral phenyl ring was not tolerated in the reaction.

**Scheme 15:**
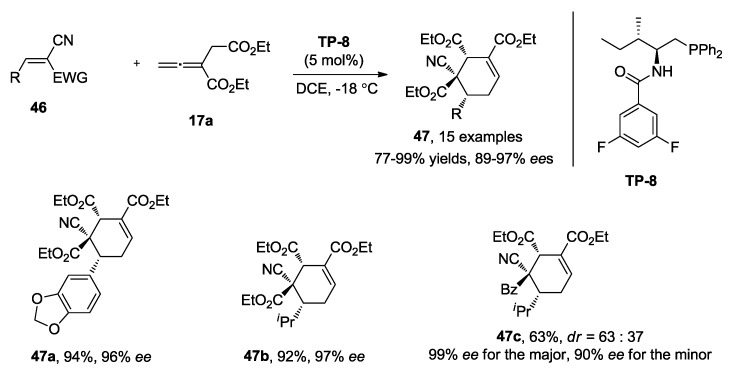
Asymmetric [4+2] cycloaddition of α-alkylallenoates with 1-cyano-1-ester olefins [53].

At the same time, by employing *l*-isoleucine-derived LB-BA bifunctional chiral tertiary phosphine compound **TP**-**8** as the catalyst, Zhao and coworkers successfully achieved the asymmetric [4+2] cycloadditions of 2-alkyl substituted allenoates with 2-cyano acrylate derived activated olefins (Scheme 15) [53]. In addition to the frequently-used aryl aldehydes-derived alkenes, aliphatic aldehyde-derived olefin (**46**, R = aliphatic) was also applied to such a stereoselective transformation for the first time, and the corresponding isopropyl cyclohexene **47b** could be obtained in 92% yield with 97% *ee*.

**Scheme 16:**
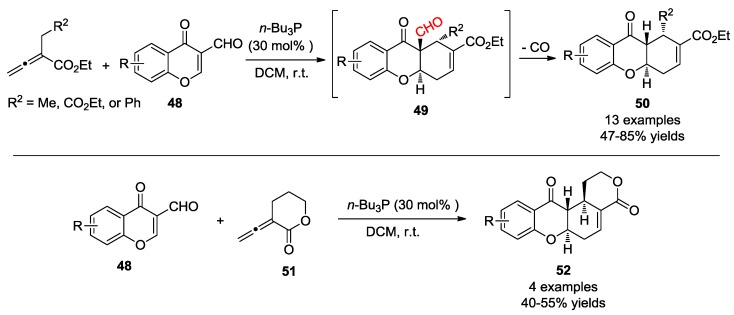
[4+2] cycloaddition of α-alkyl allenoates with chromone derivatives [54].

In recent years, some electron-deficient olefins that are embodied in novel skeleton scaffolds have been developed as dipolarophiles as well in the phosphine-catalyzed [4+2] cycloadditions with α-alkyl allenoates. To establish an efficient and concise synthetic access to the naturally occurring tricyclic benzopyrone framework, in 2011, Kumar and coworkers applied the electron-deficient 3-formy chromone as the alkene in [4+2] annulations with zwitterion **C** (γ-addition selectivity) generated by the addition of phosphine catalyst to α-alkyl allenoates (Scheme 16) [54]. A cascade sequence of [4+2] annulation followed by a deformylation that provided the desired tricyclic cyclohexene-fused chromone rings **50** with up to three stereogenic centers as a single diastereoisomer was observed. The exocyclic allenic lactone **51** proved to be a competent 1,4-dipolar *C*_4_ synthon as well in the same [4+2] annulations, smoothly delivering the tetracyclic benzopyrones **52** in high diastereoselectivities.

**Scheme 17:**
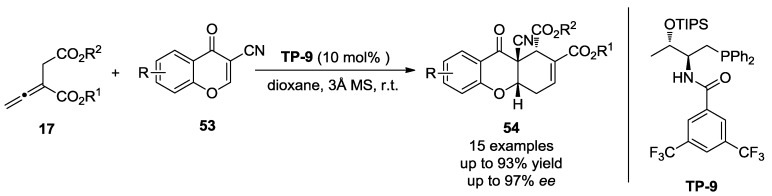
Asymmetric [4+2] cycloaddition of α-alkylallenoates with chromones [55].

In 2016, Kumar et al. realized the asymmetric catalytic version of the above [4+2] annulations (Scheme 17) [55]. Instead of the 3-formychromone, 3-cyano-chromones were employed as the activated alkenes. *l*-Threonine-derived bifunctional *N*-acylaminophosphine (**TP**-**9**) proved to be the best catalyst, mediating the [4+2] annulations with α-alkyl allenoates **17** to generate tricyclic tetrahydroxanthones **54** containing three continuous chiral centers in high yields with excellent enantioselectivitiies and moderate to high diastereoselectivities.

**Scheme 18:**
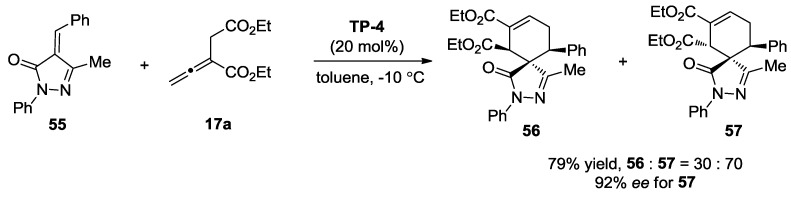
[4+2] cycloadditions of unsaturated pyrazolones with allenoates [56].

In 2015, Guo and coworkers employed the unsaturated pyrazolone **55** as a novel type of activated alkene to examine the similar phosphine-catalyzed [4+2] cycloaddition with α-substituted allenoates (Scheme 18) [56]. The moderately nucleophilic MePPh_2_ was capable of triggering the desired racemic annulation with diethyl 2-vinylidenesuccinate to afford the corresponding spiropyrazolone products in 49–99% yields as a single diastereomer. Reaction of β′-aryl-substituted allenoates with the unsaturated pyrazolone resulted in poor diastereoselectivities although the annulation proceeded smoothly to provide the corresponding cycloadducts in good to excellent yields (75–99%). Utilization of a chiral thiourea-based bifunctional phosphine **TP**-**4** (Scheme 9) as the chiral organocatalyst allowed the formation of a variety of chiral polysubstituted spiropyrazolones in moderate to excellent yields with excellent enantioselectivities and moderate to good diastereoselectivities (Scheme 18).

**Scheme 19:**
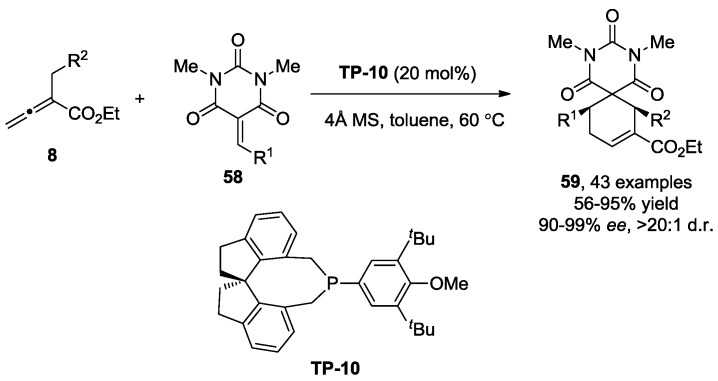
Enantioselective [4+2] cycloadditions of barbiturate-derived alkenes with α-substituted allenoates [57].

In 2016, Guo, Zhou, and co-workers found that the barbiturate-derived alkenes **58** were another type of ideal dipolar olefin in the phosphine-catalyzed asymmetric [4+2] annulation with α-alkyl allenoates (Scheme 19) [57]. The spirocyclic chiral phosphine catalyst **TP**-**10** was capable of inducing high to excellent enantioselectivities. It is noteworthy that, compared to previously reported chiral phosphine-catalyzed [4+2] annulations where only β′-alkoxy carbonyl substituted allenoate were tolerated in most cases, the allenoate scope were quite wide in this work. Not only β′-ethoxy carbonyl substitution, but non-substituted α-methyl allenoate, and α-methyl allenoates with electron-rich, -neutral, and -deficient aromatic moieties (include naphthyl substitution) on the β′-positions were also tolerated in the annulation reactions. With the adoption of this methodology, a wide variety of pharmaceutically important spirobarbiturate-cyclohexenes **59** were obtained in good to excellent yields with excellent diastereo- and enantioselectivities.

**Scheme 20:**
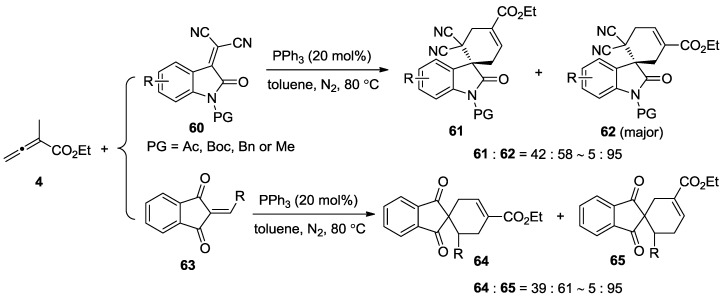
[4+2] cycloadditions of exocyclic alkenes with α-methylallenoates [58].

Although Lu’s group have reported the highly enantioselective [4+2] annulation reactions of β’-alkoxy carbonyl and β′-electron-poor aryl substituted allenoates with isatylidenemalononitrile for the synthesis of functionalized chiral 3-spirocyclohexene-2-oxindoles in 2012, reactivities of the simple, non-substituted α-methyl allenoates **4** were not examined. In 2017, Chen, He and co-workers investigated the [4+2] annulations of 2-methyl-2,3-butadienoates **4** with isatylidenemalononitrile **60** (Scheme 20) [58], and found that the expected spiroannulation could proceed smoothly to deliver two separable regioisomers **61** and **62** derived from γ- and β′-addition, respectively, when conducting the reaction at 80 °C with the use of PPh_3_ as the catalyst. Regioselectivities were unsatisfactory in most cases except for halogen-substituted *N*-Ac isatin-derived alkenes (R = Ac, **61**:**62** = 5:95). In addition, indan-1,3-dione-derived alkenes **63** were tested as well, similarly, the [4+2] annulations furnished a corresponding pair of regioisomers **64** and **65** in excellent yields with modest to good regioselectivities (**64**:**65** = 39:61~5:95). 

### 2.3. [4+2] Annulations of α-Alkyl Allenoates with Ketones

**Scheme 21:**
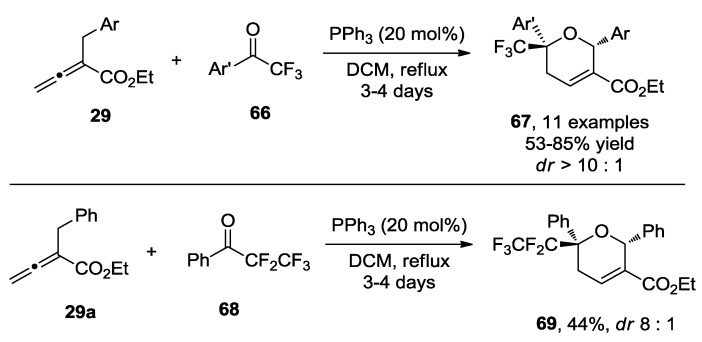
[4+2] cycloaddition of α-alkyl allenoates with trifluoromethyl (or pentafluoroethyl)aryl ketones [59].

In 2010, the group of Ye found that when trifluoromethyl(aryl)ketones **66** or pentafluoroethyl(phenyl)ketone **68** were used as the dipolarphiles, [4+2] annulations occurred to produce highly functionalized fluorinated dihydropyrans (Scheme 21) [59]. Both electron-donating and electron-withdrawing substituents were tolerated on phenyl ring in the [4+2] annulations, furnishing the desired dihydropyrans in good yields with high diastereoselectivities. Heteroaryl trifluoromethyl ketone and pentafluoroethyl(phenyl)ketone also worked to give the corresponding cycloadducts although in a slightly lower yield. However, reactions of α-methyl or α-ethoxyl carbonyl allenoates gave no or only trace corresponding [4+2] annulation product. Furthermore, other activated ketones, such as 2-oxo-2-phenylacetate, benzoyl cyanide and *N*-methylisatin, did not react with the α-benzyl allenoate under the same reaction conditions.

### 2.4. [4+3] Annulations of α-Alkyl Allenoates with Azomethine Imines

**Scheme 22:**
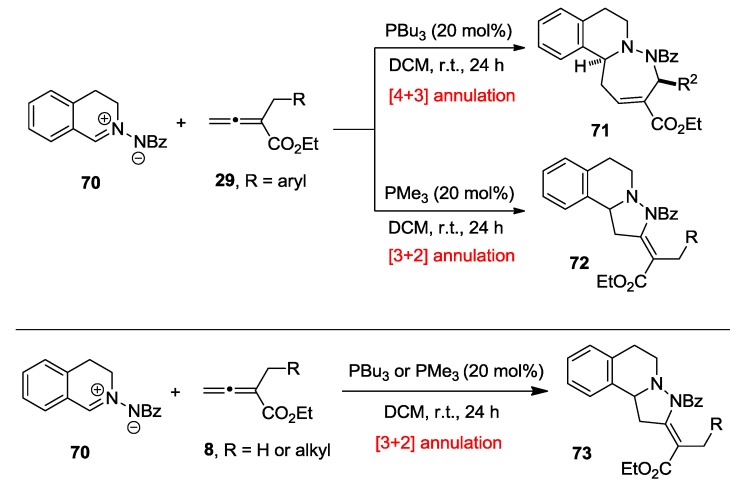
Phosphine-catalyzed [3+2] and [4+3] annulation reactions of α-substituted allenoates with azomethine imines [60].

Due to the intrinsic ring strain and competing cyclization pathways, phosphine-catalyzed cycloaddition of α-alkyl allenoates with other dipolarophiles usually lead to the formation of normal-sized 5- or 6-membered rings. Despite its great efficiency in construction of 5- or 6-membered rings, phosphine-catalyzed preparation of medium-sized rings was elusive until in 2012. Guo, Zhong, Kwon and coworkers described phosphine-catalyzed [3+2] and [4+3] annulation reactions of α-substituted allenoates with *C*,*N*-cyclic azomethine imines for the synthesis of a variety of pharmaceutically important tetrahydroisoquinoline derivatives (Scheme 22) [60]. Interestingly, they found that the nucleophilic phosphine catalyst together with the allenoate substrates had a great influence on the chemoselectivity of the annulation reaction. Generally, for the β′-aryl allenoates **29**, employment of strongly nucleophilic PBu_3_ as the catalyst mainly led to [4+3] cyclization pathways, while in the presence of PMe_3_ thermodynamically favored [3+2] annulate pathway was predominate (Scheme 22, up). However, under the catalysis of either PBu_3_ or PMe_3_, the reactions of α-alkyl allenoates **8** underwent the [3+2] cyclization process exclusively, giving only very small amounts (<1% in most cases) of the [4+3] cycloadducts (Scheme 22, bottom).

**Scheme 23:**
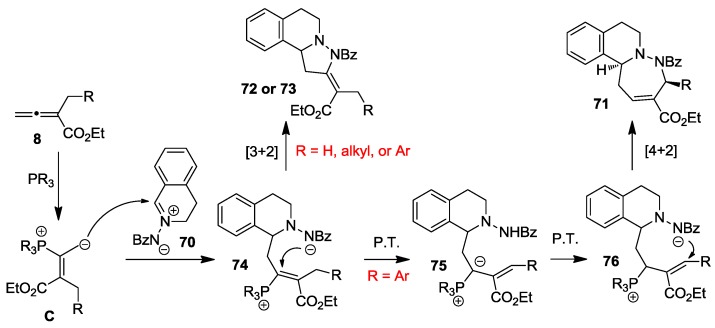
Plausible mechanism for the phosphine-catalyzed [3+2] and [4+3] annulation reactions of azomethine Imines [60].

Based on the previous reported [4+2] annulations of α-substituted allenoates, they depicted the plausible mechanism for the 1,3-dipolar cycloadditions as in Scheme 23: nucleophilic addition of the phosphine catalyst to the β-carbon of the allenoate generated the zwitterionic intermediate **C**, which subsequently attacked the azomethine imine **70** through the γ-carbon to form the zwitterionic intermediate **74**. 5-*exo* cyclization followed by a β-elimination of the phosphine catalyst afforded the [3+2] annulated product **72** or **73**. On the other hand, when R is an aryl group, a favorable sequence of proton transfer and equilibration took place, which led to isomerization of **74** to **75** due to the more acidic nature of the β′-carbon. After a further proton transfer process, 7-*endo* cyclization occurred. Expulsion of the catalyst PBu_3_ furnished the [4+3] cycloaddition product **71**.

**Scheme 24:**
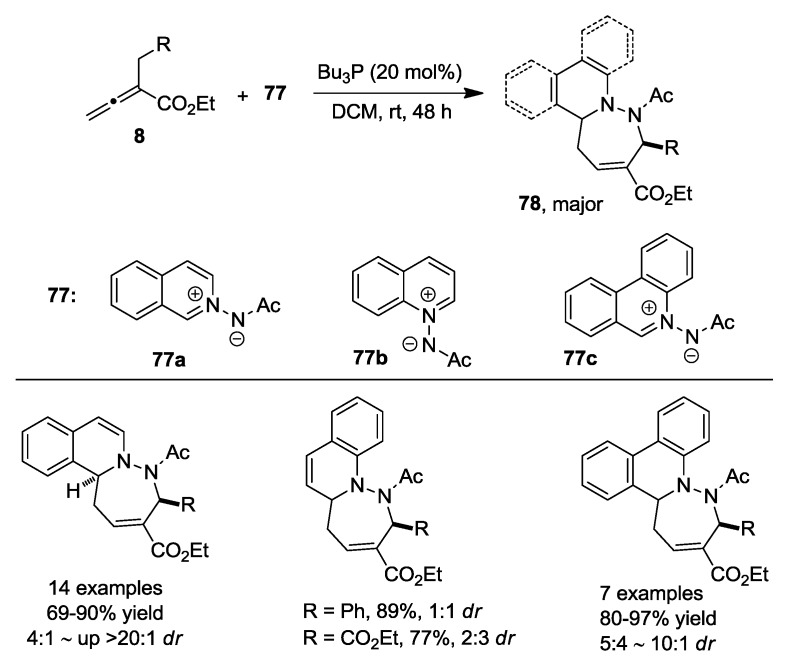
The [4+3] cycloadditions of α-substituted allenoates with azomethine imines [61].

Guo and coworkers later found that the PBu_3_-catalyzed annulation of α-substituted allenoates with *C*,*N*-cyclic aromatic azomethine imines **77**, including *N*-acetyliminoisoquinolinium betaine (**77a**), *N*-acetyliminoquinolinium betaine (**77b**), and *N*-acetyliminophenanthridinium betaine (**77c**), exclusively produced the [4+3] cycloadducts **78**, providing dinitrogen-fused heterocyclic compounds in moderate to excellent yields (Scheme 24) [61]. Both alkyl and ethoxy carbonyl, and methylene aryl groups with different electronic substitution patterns were compatible at the α-position of the allenoates in the [4+3] annulations. Compared with the initial report, this catalytic [4+3] cycloaddition process was a practical synthetic method for biologically important heterocycles, suggesting immense synthetic utility. 

**Scheme 25:**
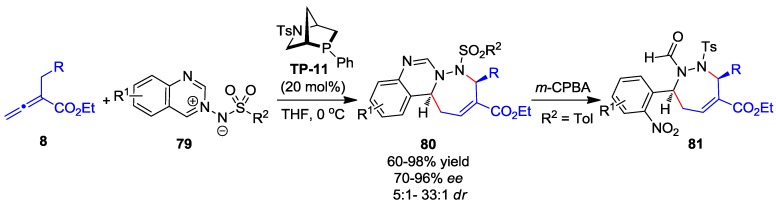
Phosphine-catalyzed enantioselective [4+3] cycloadditions of α-substituted allenoates with azomethine imines [62].

In their following research, the enantioselective version of the phosphine-catalyzed [4+3] cycloaddition of α-substituted allenoates with *C*,*N*-cyclic azomethine imines was realized for the first time (Scheme 25) [62]. The commercially available Kwon phosphine **TP**-**11** was identified as the optimal catalyst, mediating the asymmetric [4+3] cycloaddition to produce the seven-membered ring-fused quinazoline-based tricyclic heterocycles **80** in high to excellent yields with high to excellent enantioselectivities and mostly excellent diastereoselectivities. The substrate scope of both the allenoate and the azomethine imine was rather wide. Not only various allenoates bearing different electronic properties of substituents on the benzene ring of the β′-aryl allenoates but also β′-carboxylate- or alkyl-substituted allenoates were compatible in the reaction. The obtained chiral tricyclic adducts **80** could be easily transformed into monocyclic diazepines **81** which frequently show significant biological activities in pharmaceutical chemistry by an oxidation-ring opening procedure.

## 3. [4+2] Annulations of δ-Aryl Allenoates with Activated Olefins

**Scheme 26:**
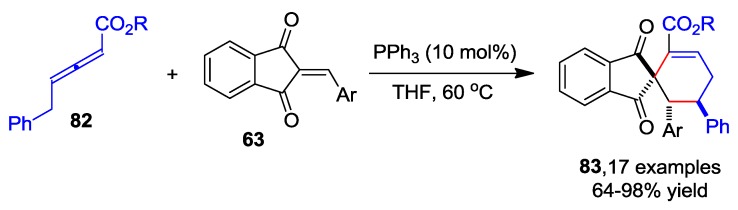
[4+2] annulation of γ-benzyl allenoates with indan-1,3-dione-derived alkenes [63].

**Scheme 27:**
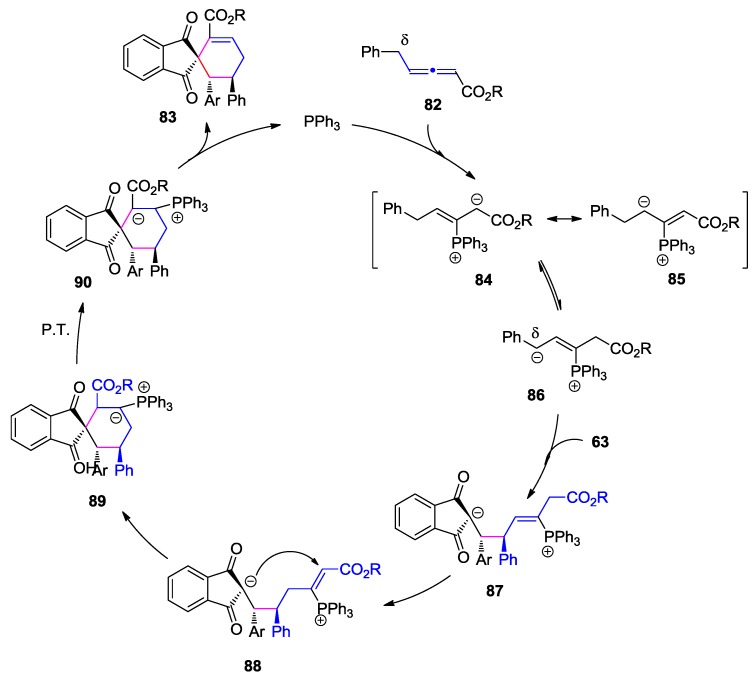
Proposed mechanisms for the [4+2] annulation of γ-benzyl allenoates with indan-1,3-dione-derived alkenes [63].

In 2013, the group of Huang disclosed an unprecedented phosphine-catalyzed [4+2] annulation of *γ*-substituted allenoates **82** with 2-arylidene-1*H*-indene-1,3(2*H*)-diones (Scheme 26) [63]. Differently from the previously reported annulation reactions that *γ*-substituent allenoates had participated in before [64,65,66,67,68], the *γ*-benzyl allenoates firstly serves as a new type of *C*_4_ synthon in this novel [4+2] cyclization process. With this method, highly substituted spiro-[4.5]-dec-6-ene skeletons **83** could be powerfully constructed in excellent yields with complete regioselectivity and high diastereoselectivity. The mechanism of this [4+2] annulation process was proposed as below in Scheme 27: firstly, nucleophilic addition of the phosphine catalyst on the *γ*-benzyl allenoate generated a pair of resonant 1,3-dipolar zwitterionic intermediates **84** and **85**. A subsequent proton transfer step gave rise to the transient allylic carbanion **86** where the negative charge was located on the δ-position of the allenoate. Michael addition of the allylic carbanion **86** to the alkene substrate **63** afforded intermediate **87**. A consecutive proton shift enabled the formation of intermediate **88**. Finally, an intramolecular umpolung addition followed by proton transfer and elimination of the phosphine catalyst furnished the desired spirocyclohexene product **83** and regenerated the catalyst.

**Scheme 28:**
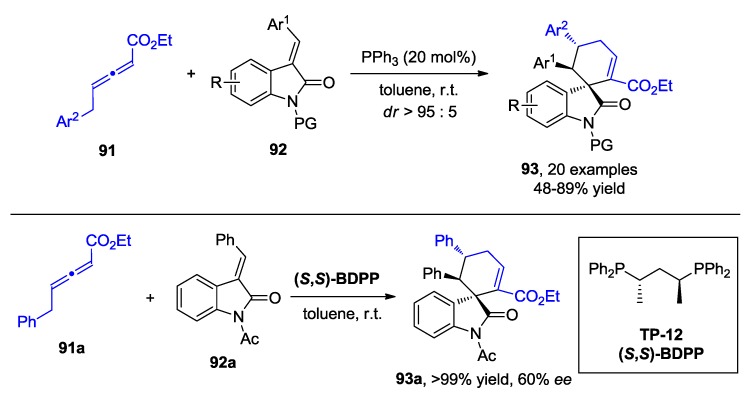
[4+2] annulation of δ-aryl-substituted penta-2,3-dienoates with 3-arylideneoxindoles [69].

At the same time, a similar phosphine-catalyzed [4+2] cycloadditions of δ-aryl-substituted penta-2,3-dienoates with 3-arylidene oxindoles **92** were also reported by Marinetti, Voituriez and coworkers (Scheme 28) [69]. Under the catalysis of PPh_3_, a wide scope of substrates were tolerated in the [4+2] annulations, providing a broad range of functionalized spiro-cyclohexene oxindoles containing three contiguous stereogenic centres in 48–89% yields with efficient stereoselective control in most cases (*dr* > 9:1 in most cases). A preliminary asymmetric catalytic study about this reaction revealed that (*S*,*S*)-2,4-bis-(diphenylphosphino)pentane ((***S*,*S***)-**BDPP, TP**-**12**) could promote the cyclization process to afford the expected spirocyclic oxindole in moderate enantioselectivity (60% *ee* for **93a**).

## 4. [4+4] Annulations of α-Methylallene Ketones with α,β-Unsaturated Imines

**Scheme 29:**
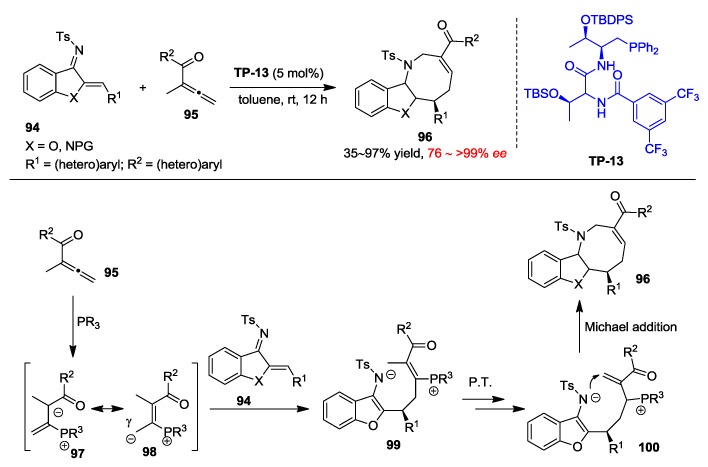
Enantioselective phosphine-catalyzed formal [4+4] annulation reaction [70].

In 2017, Lu, Ullah and co-workers disclosed the first example of a phosphine-catalyzed cycloaddition for the synthesis of eight-membered rings, achieving an enantioselective phosphine-catalyzed [4+4] annulation reaction of α-methyl allene ketones with *α*,*β*-unsaturated imines (Scheme 29) [70]. The dipeptide *l*-Thr-*l*-Thr-derived bifunctional phosphine catalyst **TP**-**13** was found to mediate the asymmetric [4+4] cycloadditions to produce eight-membered azocine in excellent yields with excellent enantioselectivities (>98% *ee* in most cases). A wide range of aurone- or aza-aurone-derived *α*,*β*-unsaturated imines bearing different aromatic moieties were compatible in the reactions, affording the corresponding benzofuran- or indole-fused azocines respectively. A strong electron-withdrawing protecting group on the nitrogen atom of the imine was required, as sulfonamide was adequate to involve into the expected [4+4] cycloadditions while imines with an alkyl or aryl group on the *N* atoms were inert under the same reaction conditions. Employment of terminal α-methyl allene ketones was another crucial point for the success of the reactions because either the similar α-substituted allenoates or γ-substituted allene ketones failed to react with the *α*,*β*-unsaturated imines. The mechanism proposed for the [4+4] cycloadditions was similar to those for the [4C+X] annulations of α-substituted allenoates: firstly, nucleophilic addition of the phosphine catalyst to the α-methyl allene ketones **95** led to the formation of a pair of resonant zwitterionic intermediates **97** and **98**. Then, γ-addition of the phosphonium enolate **98** to the α,β-unsaturated imines **94** afforded intermediate **99**. Subsequently, a proton shift enabled alkeneisomerization to give **100**. Finally, cyclization and release of the phosphine catalyst gave rise to the eight-membered product **96**. The authors explained that the steric hindrance of the NTs anion avoided the [4+2] annulation pathways. 

## 5. [4+X] Annulations of β′-Acetoxy Allenoates

**Scheme 30:**
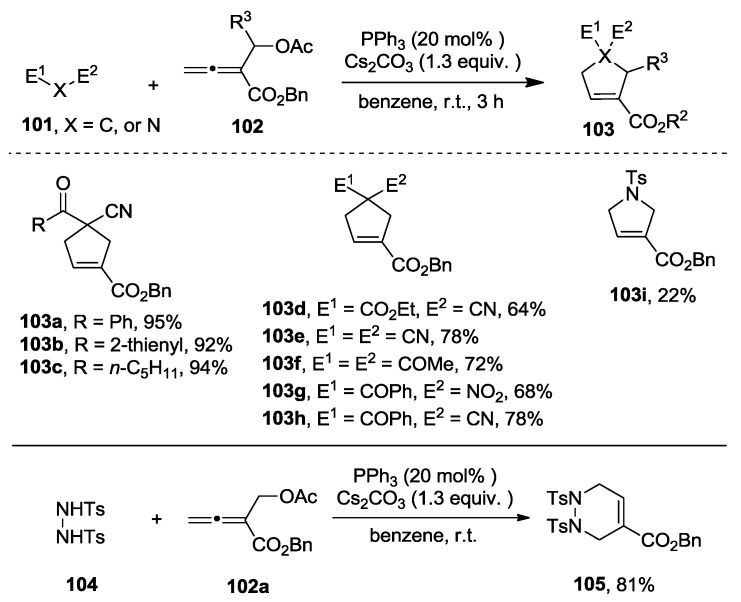
Phosphine-catalyzed [4C+X] annulations of 1,4-biselectrophiles [71].

Generally, the zwitterionic intermediates derived from nucleophilic addition of the phosphine catalyst to the allenoates are nucleophilic to react with various electrophiles, but a remarkable reversal of polarity was witnessed after elimination of a negative charged group of the zwitterion. In 2010, Tong’s group innovatively introduced an acetoxy group (-OAc) at the β′-position of 2,3-butadienoate as a leaving group, enabling it to serve as a versatile 1,4-biselectrophilic precursor and participate in [4C+X] annulations with 1,n-bisnucleophiles under the catalysis of triphosphine catalyst (Scheme 30) [71]. [4+1] annulations took place when α-cyanoketones were used as the other coupling reactant, providing functionalized cyclopentenes in good to excellent yields. Other similar one-carbon bisnucleophiles, such as α-cyano ester, malononitrile, α-nitryl ketones, 1,3-dicarbonyl compounds, as well as heteroatomatic bisnucloephile, such as tosyl amide, could also be employed in the [4+1] annulations, albeit the 2,5-dihydropyrrole **103i** was obtained in a much lower yield (22%) when tosyl amide (TsNH_2_) was utilized. *N*-Ts hydrazide **104** could also serve as a type of two-atomatic 1,2-bisnucleophiles, participating in analogous [4+2] annulation with 2-(acetoxymethyl) buta-2,3-dienoate **102a** to produce the corresponding tetrahydropyridazine derivative **105** in 81% yield. 

**Scheme 31:**
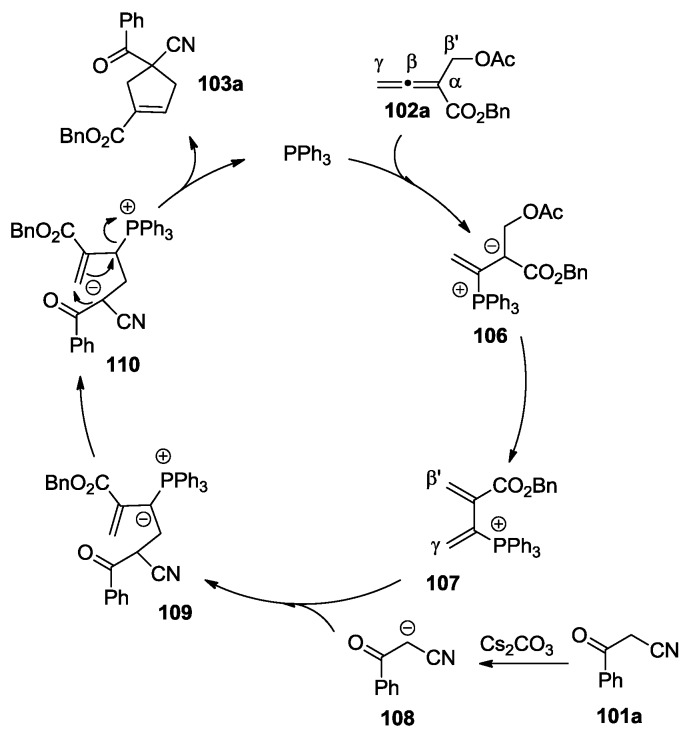
Proposed mechanism for the [4+1] annulation1,4-biselectrophiles [71].

Taking the [4+1] annulation of 2-(acetoxymethyl)buta-2,3-dienoate **102a** with 3-oxo-phenyl-propanenitrile **101a** as an example, the proposed mechanism was as depicted in Scheme 31. Firstly, nucleophilic addition of PPh_3_ to 2-(acetoxymethyl)buta-2,3-dienoate **102a** formed the zwitterionic intermediate **106**, whose negative charge on the α-position promoted a subsequent 1,2-elimination of an acetate anion (AcO^−^) to generate the 1,4-biselectrophilic intermediate **107**. Then, after deprotonation by the base, the 1,1-bisnucleophile **101a** became a carbanionic intermediate **108**, which attacked the *γ*-carbon of 1,4-biselectrophilic intermediate **107** to provide phosphonium ylide **109.** Thirdly, the original α-position of 1,4-bisnucleophile underwent a deprotonation again via intramolecular proton shift and thus resulted in the formation of intermediate **110.** Finally, conjugate addition and recycling of the phosphine catalyst furnished the [4+1] cycloadduct **103a**.

**Scheme 32:**
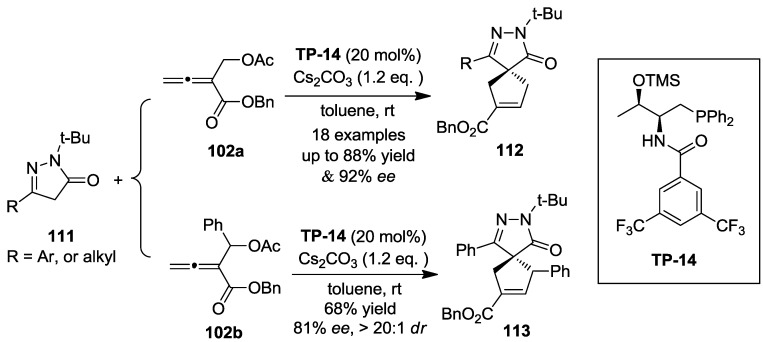
Asymmetric synthesis of functionalized spiropyrazolones [72].

In 2014, highly enantioselective [4+1] annulation of the 1,4-biselectrophiles with pyrazolone **111** was realized by the group of Lu for the first time (Scheme 32) [72]. *l*-threonine-derived *O*-silylated bifunctional phosphine catalyst **TP**-**14** was the optimal catalyst to promote the expected [4+1] annulation reaction to generate a series of chiral spiropyrazolones **112** that have potential biological activity in good yields with good to high enantioselectivities. 

**Scheme 33:**
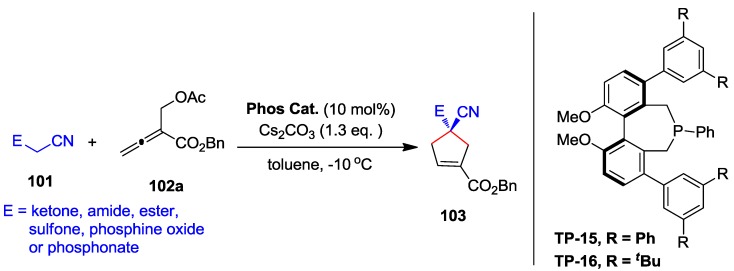
Enantioselective [4+1] annulations to synthesize functionalized cyclopentenes [73].

**Scheme 34:**
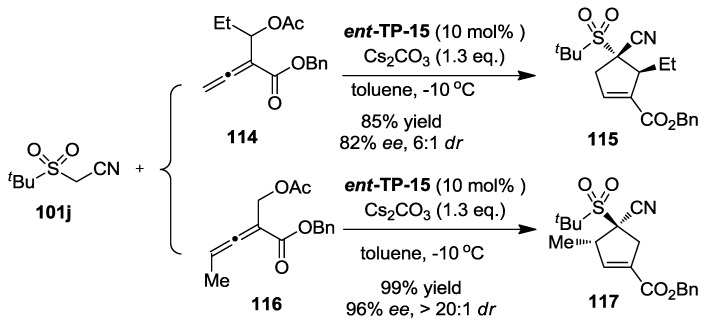
Enantioselective [4+1] annulations to synthesize functionalized cyclopentenes with two consecutive stereocenters [73].

Almost at the same time, the asymmetric [4+1] annulations of *β*′-acetoxy allenoates were reported by Fu and co-workers (Scheme 33) [73]. The biphenyl-derived axially chiral phosphine was used as the catalyst and a wide array of α-cyano compounds, such as α-cyano ketones, amides, esters, sulfones, phosphine oxides and phosphonates, were employed as the nucleophiles. A wide variety of functionalized cyclopentenes **103** bearing non-spirocyclic, fully substituted stereocenters (either all-carbon or heteroatom-substituted (sulfur and phosphorus)) were assembled in high yields with good to high enantiomeric excesses. Cyclopentenes bearing two consecutive stereocenters could also be generated under the catalytic system in good stereoselectivities when β′- or γ-substituted β′-acetoxy allenoates were employed (Scheme 34). Furthermore, a mechanistic study revealed that the turnover-limiting step occurs after the addition of the chiral phosphine to the allenoate, and likely after the addition of the second coupling partner as well.

**Scheme 35:**
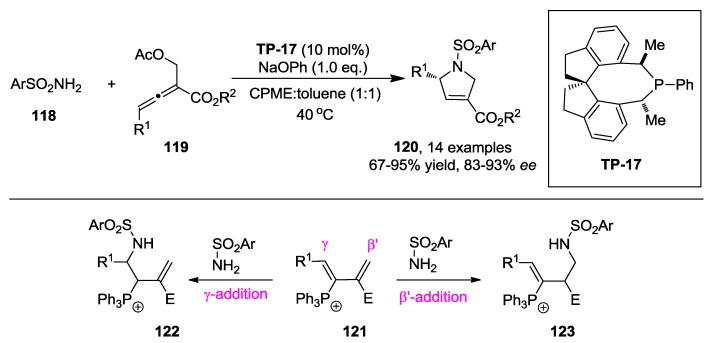
Enantioselective [4+1] annulations of amines with γ-substituted 2-(acetoxymethyl)buta-2,3-dienoate [74].

One example of [4+1] cycloaddition of 2-(acetoxymethyl)buta-2,3-dienoate **102** with nitrogen nucleophile (TsNH_2_) was reported in Tong’s work, but the reaction afforded an achiral 2,5-dihydropyrrole **103i** in low yield (Scheme 30) [71]. In 2015, the group of Fu disclosed an asymmetric [4+1] cycloaddition of *γ*-substituted 2-(acetoxymethyl)buta-2,3-dienoate **102** with sulfonamides (Scheme 35) [74]. With the aid of a novel chiral spirophosphine catalyst **TP**-**17**, they achieved the objectives for constructing an array of enantioenriched 2,5-dihydropyrrole products **120** in high yields (67–95%) with excellent enantioselectivities (83–93% *ee*) in which the newly formed stereocenter emanated from the *γ*-carbon of the racemic allenes **119**. Control experiments proved that both β′- and *γ*-addition pathways were feasible when the sulfonamide **118** coupled with the 1,4-biselectrophilic intermediates **121**.

**Scheme 36:**
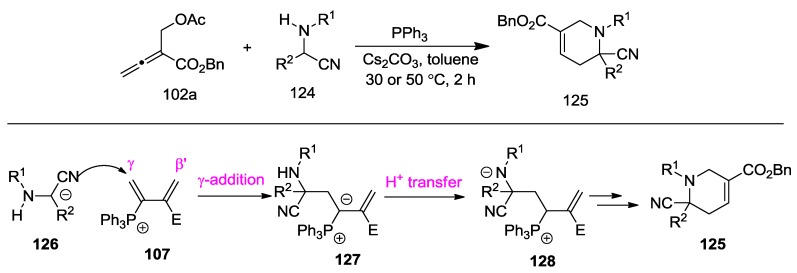
Phosphine-catalyzed [4+2] annulations of α-aminonitriles with 2-(acetoxymethyl)buta-2,3-dienoate [75].

In 2016, α-aminonitriles **124** were employed as *C*,*N*-bisnucleophiles to react with 2-(acetoxymethyl)buta-2,3-dienoates by Liao, Zhang and coworkers (Scheme 36) [75]. Under the catalysis of PPh_3_, the [4+2] cycloaddition of a wide range of α-aminonitriles **124** proceeded smoothly to furnish the corresponding poly-substituted tetrahydropyridines **125** which include a quaternary carbon stereocenter in moderate to good yields. Although a variety of α-aminonitriles were compatible in the annulation, reaction of *N*-Ts protected α-aminonitrile did not form the same [4+2] cycloadduct. Concerning the mechanism, the authors proposed a firstly γ-addition of the nucleophilic carbanion to the 1,4-biselectrophile before proton transfer and cyclization.

## 6. [4+2] Annulations of δ-Acetoxy Allenoates

**Scheme 37:**
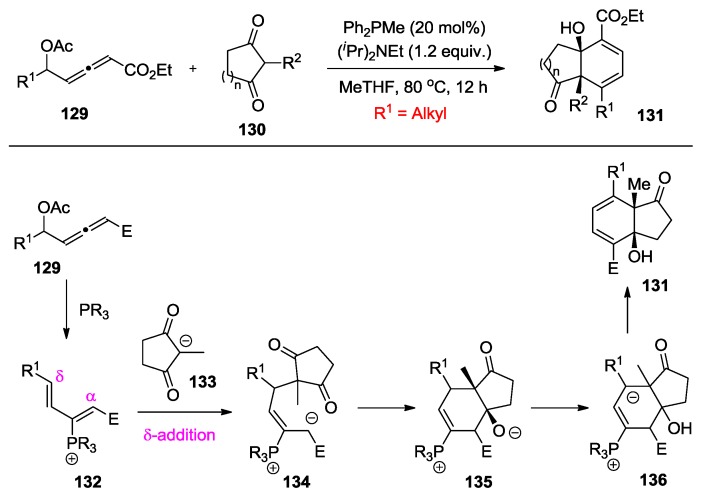
Phosphine-catalyzed [4+2] annulations of δ-acetoxy allenoates and 1,3-diketones and related mechanisms [76].

**Scheme 38:**
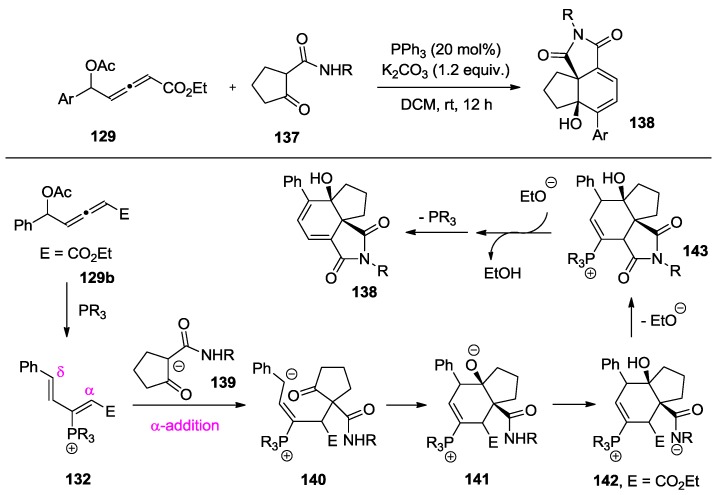
Phosphine-catalyzed [4+2] annulations of δ-acetoxy allenoates and cyclic β-carbonyl amides [76].

In 2017, the group of Tong disclosed a novel type of phosphine-catalyzed, substrate-dependent [4+2] annulations of *δ*-acetoxy allenoates with ketones (Scheme 37) [76]. Under the optimized reaction conditions (running the reaction in MeTHF at 80 °C with the use of PPhMe_2_ as the catalyst and *^i^*Pr_2_NEt as the base), the [4+2] cycloadditions of *δ*-acetoxy allenoates **129** with 2-substituted cyclic 1,3-diones **130** mainly formed the functional group rich and structurally complex 1,3-cyclohexadiene compounds **131**. However, *δ*-acetoxy allenoates bearing an aryl group at the δ-position seemed unsuitable for the above [4+2] annulations, as reaction with the diketone **130** led to formation of a complex mixture under the same reaction conditions. With their continued efforts, soon after, they found that cyclic β-carbonyl amides **137** were suitable annulation partners to react with the allenoates **129**. Interestingly, with the use of PPh_3_ as the catalyst and K_2_CO_3_ as the base when running the reaction in DCM at room temperature, reaction of the cyclic β-carbonyl amides **137** with *δ*-aryl-substituted *δ*-acetoxy allenoates involved in a cascade “[4+2] annulation/cyclic imide formation” process, furnishing a novel array of structurally more complicated tricyclic products **138** in good yields (50–94%) (Scheme 38).

With regard to mechanisms, initiation of the reaction was very similar to that of the β′-acetoxy allenoates, that is, firstly cationic intermediate **132**, which has been proven to be a good 1,4-biselectrophile toward annulations with bisnucleophiles, was formed through an “addition−elimination” sequence. However, when 2-substituted cyclic 1,3-diones were utilized as the annulation partner, nucleophilic addition of the corresponding carbanionic diketone intermediate **133** to the 1,4-biselectrophile **132** preferred δ-addition, allowing for the formation of phosphonium enolate **134** (Scheme 37, bottom). Then, intramolecular nucleophilic attacked of the α-carbanion to the ketone afforded alkoxide intermediate **135**. Intramolecular proton transfer and elimination of the phosphine catalyst ultimately led to the production of 1,3-cyclohexadiene cycloadduct **131**.

In stark contrast, the enolate of cyclic β-carbonyl amides **139** preferentially attacked the α-position of 1,4-biselectrophilic intermediate **132** to give the intermediate **140**. Then, nucleophilic addition of the δ-carbon anion to the ketone resulted in cyclization and the formation of alkoxide intermediate **141**, which would abstract proton from the amide. Subsequently, the amide attacked the spatially adjacent ester and kicked away EtO^−^, resulting in the formation of cyclic imide **143**. Finally, with the assistance of the released strong base EtO^−^, deprotonation, proton transfer and elimination of the phosphine catalyst successively happened, thus yielding the tricyclic 1,3-cyclohexadiene products **138** (Scheme 38, bottom). 

**Scheme 39:**
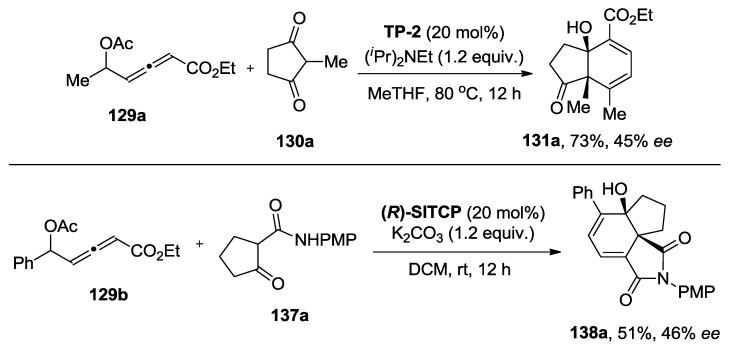
Asymmetric phosphine-catalyzed [4+2] annulations of δ-acetoxy allenoates [76].

Preliminary catalytic asymmetric investigation of the two types of [4+2] annulations of *δ*-acetoxy allenoates was also conducted, and the results indicated that approximately 45% *ee* were obtained for the two reactions at this stage (Scheme 39).

## 7. [4+X] Annulations of Electron-Deficient 1,3-Dienes

**Scheme 40:**
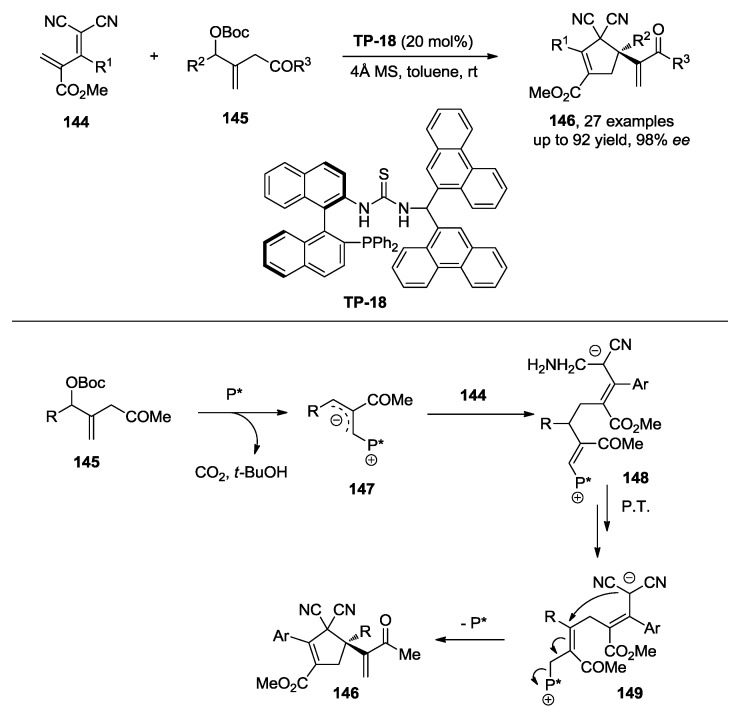
[4+1] annulation of electron-deficient 1,3-diene with MBH carbonates [77].

In 2012, a novel phosphine-catalyzed asymmetric [4+1] annulation of MBH carbonates **145** with dicyano-2-methylenebut-3-enoates **144** was developed by Shi and coworkers (Scheme 40) [77]. Catalyst screening and reaction conditions optimization revealed that the multifunctional chiral phosphine catalyst **TP**-**18** bearing an axially chiral binaphthyl scaffold was the most effective catalyst when the reaction was conducted in toluene at room temperature with 4 Å MS as the additive. A wide array of highly functionalized cyclopentenes **146** bearing an all-carbon quaternary stereogenic center was efficiently synthesized in moderate to good yields with excellent enantioselectivities. Based on previous correlative research works, a plausible reaction mechanism was proposed as: initially, an addition–elimination–deprotonation sequential processes resulted in the formation the 1,1-dipolar (or 1,3-dipolar in other transfermations) phosphonium ylide **147**; conjugate addition of phosphorus ylide **147** to the activated 1,3-dienes **144** at the *C*_1_-terminal position furnished intermediate **148**; then, a consecutive proton transfer took place to cause alkene isomerization; and, finally, an intramolecular Michael addition followed by elimination of the phosphine catalyst gave rise to the [4+1] cycloadduct **146**. 

**Scheme 41:**
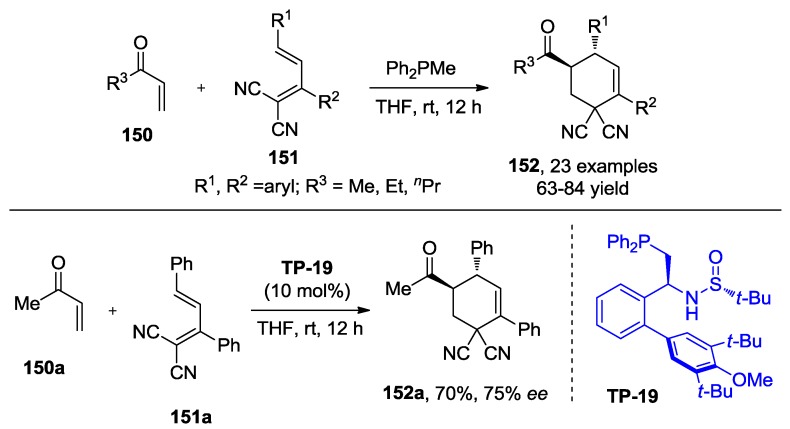
[4+2] annulation of electron-deficient 1,3-diene with alkyl vinyl ketone [78].

Although Diels–Alder reaction are prevalent methods to assemble six-membered cyclohexenes, the sensitivity to steric hindrance and the strict requisite for proper electron-property usually limited the application. Delightedly, triphosphine initiated dipolar cycloadditions can supplement its deficiency. In 2018, Zhang’s group firstly developed a phosphine-catalyzed [4+2] annulation of electron-deficient diene with the alkyl vinyl ketone that hardly occur in Diels–Alder reactions (Scheme 41) [78]. The [4+2] cycloaddition of a series of β,δ-diaryl-substituted electron-deficient dienes **151** with alkyl vinyl ketones **150** were carried out in THF at room temperature with the use of Ph_2_PMe (10 mol%) as the catalyst, which proceeded smoothly to produce functionalized cyclohexenes **152** in moderate to good yields with excellent diastereoselectivities. However, *β*-alkyl-substituted electron-deficient dienes and other electron-deficient olefins except alkyl vinyl ketone, such as acrolein and phenyl ketone, were incompatible in the [4+2] cycloadditions. Preliminary catalytic asymmetric investigation revealed that the use of Peng-Phos (**TP**-**19**) developed by their group afforded the chiral product **152a** in 70% yield with 75% *ee*.

## 8. [4+2] Spiroannulations of Cyclobutenones

**Scheme 42:**
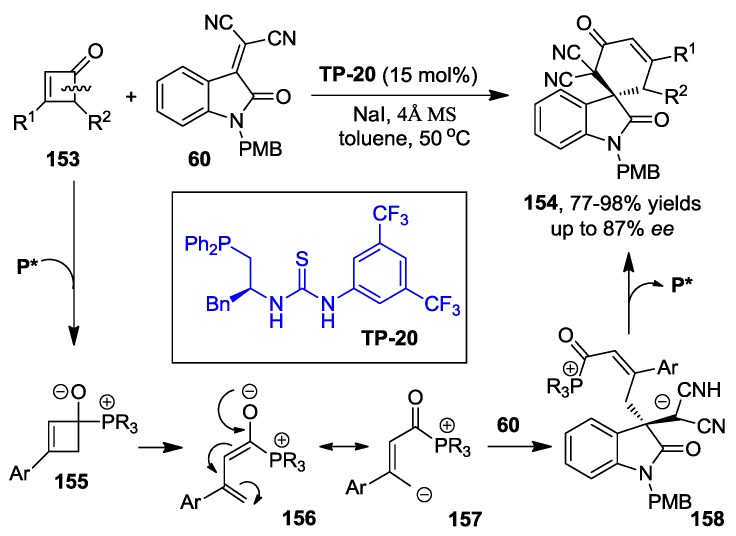
Enantioselective [4+2] cycloaddition of cyclobutenones with isatylidenemalononitriles [79].

In 2015, the group of Zhang firstly exploited cyclobutenones as a novel type of 1,4-dipolar precursor in phosphine catalysis (Scheme 42) [79]. Under the catalysis of phenylanine-derived LB-BA bifunctional chiral phosphine catalyst **TP**-**20**, the enantioselective 1,4-dipolar spiroannulations of cyclobutenones **153** with isatylidenemalononitriles **60** produced the enantioenriched 3-spiro-cyclohexenone-2-oxindoles **154** in good to excellent yields with up to 87% *ee*. Generally speaking, the organo-phosphine catalyzed reaction processes were usually initiated by the conjugate addition of the strong nucleophilic but weak basic phosphine atom to the C-C multiple bonds, such as alkenes, allenes and alkynes, but Zhang and coworkers proposed another unique and unclassical activation mode, that is, the nucleophilic phosphine catalyst initially chemoselectively attacked the carbonyl group of cyclobutenones to entice the reaction. The subsequently formed oxygen anion of intermediate **155** promoted C-C single bond activation and cleavage of the small ring to furnish a novel type of vinyl-enolate-based 1,4-dipoles **156** and **157**. Nucleophilic addition of the 1,4-dipoles to the isatylidenemalononitrile **60** formed intermediate **158**. Finally, cyclization accompanied with the regeneration of phosphine catalyst produced the spirocyclic products **154**. 

## 9. Other Miscellaneous [4C+X] Annulations

**Scheme 43:**
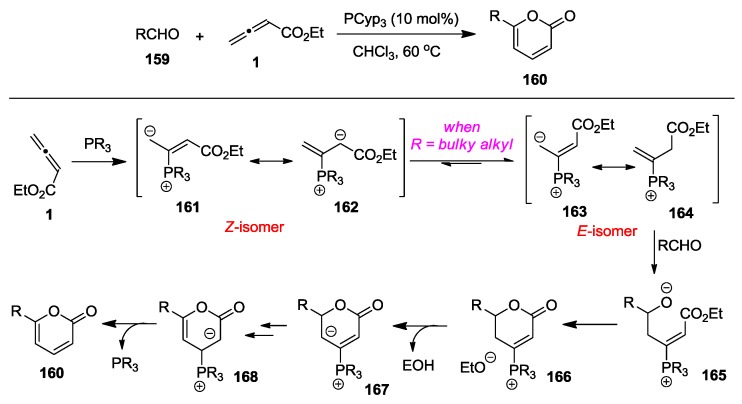
Phosphine-catalyzed [4+2] cycloaddition of allenoates with aldehydes [80].

Usually, allenoates serve as *C*_2_ or *C*_3_ synthons in the phosphine-catalyzed cycloadditions, but in 2005, Kwon and coworkers demonstrated that the reaction of allenoates with aldehydes could also proceed via [4+2] cycloaddition process when bulky trialkyl phosphine catalysts were employed (Scheme 43) [80]. The subtlety of this unique chemoselectivity is that, when an allenoate was added by a phosphine catalyst, the use of sterically hindered trialkylphosphines, such as tricyclopentyl phosphine, facilitated the shift of equilibrium from the *Z*- toward *E*-isomeric zwitterionic intermediate to minimize the steric interaction between the phosphonium moiety and the ethoxy carbonyl group. Upon addition to the other aldehyde at the γ-carbon, the consequently formed alkoxide was in close proximity to the ester group, which would promote intramolecular nucleophilic attack of the alkoxide to the ester to furnish the lactone intermediate **166**. The ejected ethoxide (EtO^−^) then acts as a base to abstract proton from the lactone intermediate, which subsequently induce a successive proton-transfer processes. Elimination of the triphosphine catalyst released the final 2-pyrone products **160**.

**Scheme 44:**
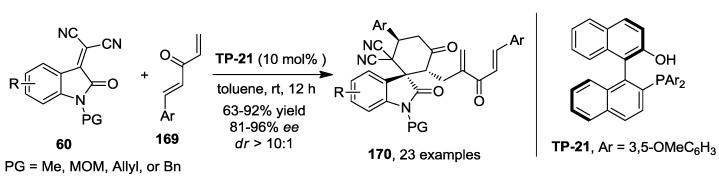
Asymmetric synthesis of functionalized spirocyclohexanoneoxindoles [81].

**Scheme 45:**
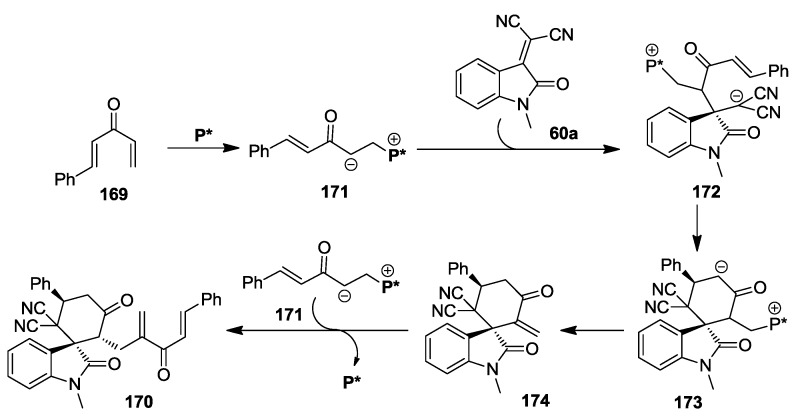
Proposed mechanism for the [4+2] tandem cyclizationof 1,4-dien-3-one with isatylidenemalononitriles [81].

In 2010, Shi, Wei and coworkers reported a highly enantioselective phosphine-catalyzed formal [4+2] tandem cyclizations between isatylidenemalononitriles **60** and 1,4-dien-3-one **169** to synthesize multi-stereogenic spirocyclicoxindoles **170** (Scheme 44) [81]. The LB-BA bifunctional chiral tertiary phosphine catalyst **TP**-**21** derived from an axially chiral binaphthyl skeleton was able to achieve the asymmetric synthesis of the multi-stereogenic spirocyclicoxindoles **170** in high yields along with excellent enantioselectivities and diastereoselectivities. The whole reaction process progressed through a tandem “Rauhut–Currier/Michael/Rauhut–Currier” reaction sequence, where two molecules of 1,4-dien-3-one **169** were involved into the reaction and were embedded in the final product **170**. Detailed reaction mechanism was proposed as below in Scheme 45: firstly, nucleophilic addition of the chiral phosphine catalyst **TP**-**21** to the vinyl ketone **169** initiated the reaction. Then, the resulted zwitterionic intermediate **171** underwent a nucleophilic attack to the isatylidenemalononitrile to produce intermediate **172**. Subsequently, an intramolecular Michael addition, followed by proton transfer and elimination of the phosphine catalyst, yielded the electronically neutral molecular intermediate **174**. As the intermediate **174** still contained an activated C-C double bond, it was hard for reaction to cease at this step, thus, another Rauhut–Currier reaction took place in the presence of the active phosphine catalyst to give the cascade product **170**.

**Scheme 46:**
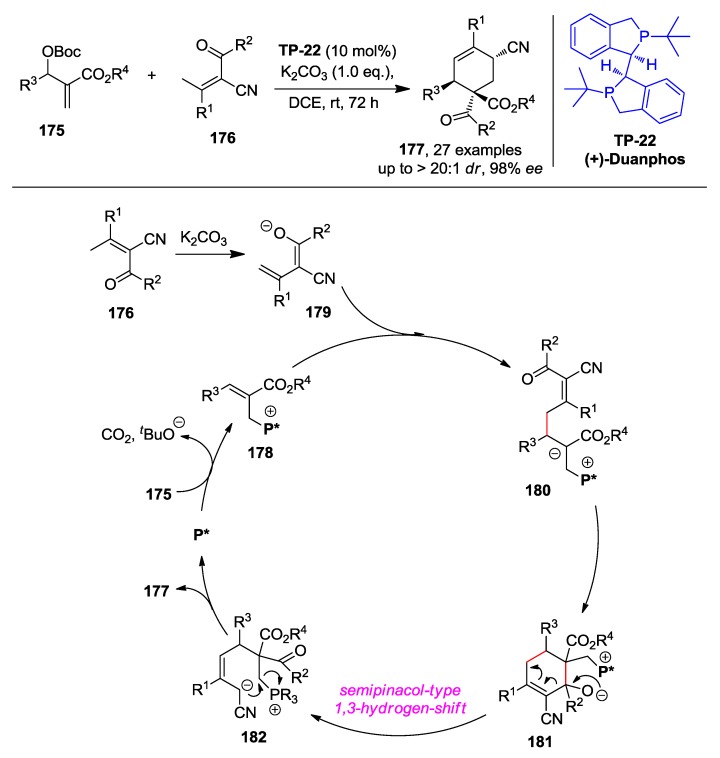
Enantioselective [4+2] cycloaddition-semipinacol-type-rearrangement [82].

Most recently, the group of Jiang developed an efficient, chiral phosphine-triggered synthetic route to access functionalized cyclohexenes via a cascade [4+2] cycloaddition/semipinacol-type rearrangement (Scheme 46) [82]. In the presence of (+)-Duanphos (**TP**-**22**), a series of 2-(acyl)but-2-enenitriles **176** were treated with MBH (Morita–Baylis–Hillman) carbonates **175** in DCE, affording the desired cyclohexene derivatives **177** in high levels of enantio- and diastereoselectivities (up to 98% *ee* and >20:1 *dr*). This reaction was initiated by the nucleophilic addition of the chiral phosphine catalyst to the MBH carbonates and a subsequent ejection of CO_2_ and *^t^*BuO^−^ to give the phosphonium intermediate **178**, which would be attacked by the incoming nucleophiles **179** that were deprotonated from the (*E*)-2-benzoyl-3-phenylbut-2-enenitriles **176**. Subsequent cyclization via intramolecular nucleophilic addition to the ketones formed the six-membered zwitterionic intermediate **181**. Finally, semipinacol-type sigmatropic 1,3-hydrogen shift occurred leading to ring-opening, which was followed by intramolecular S_N_2 substitution to regenerate the phosphine catalyst to complete the catalytic cycle and deliver the cyclohexene product **177**.

## 10. Conclusions

In summary, with the continuous efforts to explore novel *C_4_* annulation partners in the area of phosphine catalysis, many types of substrates have been successfully introduced into the [4C+X] cycloadditions. Except electron-deficient dienes and cyclobutenones, almost all others types of substrates are based on the skeleton of allenes, namely α-substituted allenoates, δ-substituted allenoates, α-methyl allene ketones, β′-acetoxy allenoates and δ-acetoxy allenoates. Despite these great studies together with the corresponding asymmetric [4C+X] annulations have been performed, there is still much to be discovered. Except for α-substituted allenoates, there are very few types of [4C+X] annulations based on the already known *C*_4_ synthons. Searching for even more extensive suitable coupling partners to cyclize with them is always highly desirable. On the other hand, finding novel *C*_4_ synthetic blocks and relevant novel annulation patterns as well as the development of the corresponding asymmetric catalytic forms are undoubtedly the permanent goals in this field due to its great efficiency to construct enantioenriched functionalized cyclic compounds. Meanwhile, some of the methodologies posed very obvious drawbacks, such as limited substrates compatibility and poor stereoselectivity, thus deep theoretical studies as well as appropriate catalyst design are also desired to address these issues. Nevertheless, with the increasing research interests thrown into the field of phosphine catalysis, we are confident that much more meaningful phosphine-catalyzed [4C+X] annulations will spring up in the next decade. We especially expect wonderful applications of these methodologies into the synthesis of natural products or the preparations of biologically important molecules in the future.

## Figures and Tables

**Table:** 

Entry	Phos Cat.	E	Yield (%)	Ee (%)
1	**TP-15**	Ph	85	94
2	**TP-15**	2-thienyl	88	90
3	**TP-15**	NPh_2_	93	91
4	**TP-15**	OMe	61	82
5	**TP-16**	SO_2_*^t^*Bu	97	94
6	**TP-16**	POPh_2_	86	84
7	**TP-16**	PO(OPh)_2_	88	87

## References

[1] Lu X., Zhang C., Xu Z. (2001). Reactions of Electron-Deficient Alkynes and Allenes under Phosphine Catalysis. Acc. Chem. Res..

[2] Ye L.-W., Zhou J., Tang Y. (2008). Phosphine-triggered synthesis of functionalized cyclic compounds. Chem. Soc. Rev..

[3] Cowen B.J., Miller S.J. (2009). Enantioselective catalysis and complexity generation from allenoates. Chem. Soc. Rev..

[4] Marinetti A., Voituriez A. (2010). Enantioselective Phosphine Organocatalysis. Synlett.

[5] Wei Y., Shi M. (2010). Multifunctional Chiral Phosphine Organocatalysts in Catalytic Asymmetric Morita-Baylis-Hillman and Related Reactions. Acc. Chem. Res..

[6] Wang S.-X., Han X.Y., Zhong F.R., Wang Y.Q., Lu Y.X. (2011). Novel Amino Acid Based Bifunctional Chiral Phosphines. Synlett.

[7] Zhao Q.-Y., Lian Z., Wei Y., Shi M. (2012). Development of asymmetric phosphine-promoted annulations of allenes with electron-deficient olefins and imines. Chem. Commun..

[8] Gomez C., Betzer J.-F., Voituriez A., Marinetti A. (2013). Phosphine Organocatalysis in the Synthesis of Natural Products and Bioactive Compounds. ChemCatChem.

[9] Xie P., Huang Y. (2013). Domino cyclization initiated by cross-rauhut-currier reactions. Eur. J. Org. Chem..

[10] Fan Y.C., Kwon O. (2013). Advances in nucleophilic phosphine catalysis of alkenes, allenes, alkynes, and MBHADs. Chem. Commun..

[11] Wei Y., Shi M. (2013). Recent Advances in Organocatalytic Asymmetric Morita−Baylis−Hillman/aza-Morita−Baylis−Hillman Reactions. Chem. Rev..

[12] Wang Z., Xu X., Kwon O. (2014). Phosphine catalysis of allenes with electrophiles. Chem. Soc. Rev..

[13] Wei Y., Shi M. (2014). Applications of Chiral Phosphine-Based Organocatalysts in Catalytic Asymmetric Reactions. Chem. Asian J..

[14] Zhou R., Liu R., Li R., He Z. (2014). Progress in Phosphine-Promoted Annulations between Two Electrophiles. Chin. J. Org. Chem..

[15] Wang T., Han X., Zhong F., Yao W., Lu Y. (2016). Amino Acid-Derived Bifunctional Phosphines for Enantioselective Transformations. Acc. Chem. Res..

[16] Li W., Zhang J. (2016). Recent developments in the synthesis and utilization of chiral b-aminophosphine derivatives as catalysts or ligands. Chem. Soc. Rev..

[17] Raghu M., Grover J., Ramasastry S.S.V. (2016). Cyclopenta[*b*]annulation of Heteroarenes by Organocatalytic γ’[C(sp^3^)-H] Functionalization of Ynones. Chem. Eur. J..

[18] Xiao Y., Guo H., Kwon O. (2016). Nucleophilic Chiral Phosphines: Powerful and Versatile Catalysts for Asymmetric Annulations. Aldrichimica Acta.

[19] Sun Y.-L., Wei Y., Shi M. (2017). Applications of Chiral Thiourea-Amine/Phosphine Organocatalysts in Catalytic Asymmetric Reactions. ChemCatChem.

[20] Wei Y., Shi M. (2017). Lu’s [3+2] cycloaddition of allenes with electrophiles: Discovery, development and synthetic application. Org. Chem. Front..

[21] Ni H., Chan W.-L., Lu Y. (2018). Phosphine-Catalyzed Asymmetric Organic Reactions. Chem. Rev..

[22] Guo H., Fan Y.C., Sun Z., Wu Y., Kwon O. (2018). Phosphine Organocatalysis. Chem. Rev..

[23] Zhang C., Lu X. (1995). Phosphine-Catalyzed Cycloaddition of 2,3-Butadienoates or 2-Butynoates with Electron-Deficient Olefins. A Novel [3+2] Annulation Approach to Cyclopentenes. J. Org. Chem..

[24] Zhang Q., Meng L.-G., Zhang J., Wang L. (2015). DMAP-Catalyzed [2+4] Cycloadditions of Allenoates with N-Acyldiazenes: Direct Method to 1,3,4-Oxadiazine Derivatives. Org. Lett..

[25] Zhu X.-F., Henry C.E., Wang J., Dudding T., Kwon O. (2005). Phosphine-Catalyzed Synthesis of 1,3-Dioxan-4-ylidenes. Org. Lett..

[26] Lu Z., Zheng S., Zhang X., Lu X. (2008). An Unexpected Phosphine-Catalyzed [3+2] Annulation. Synthesis of Highly Functionalized Cyclopentenes. Org. Lett..

[27] Fu J., Na Z., Peng B., Uttamchandani M., Yao S.Q. (2016). Accelerated cellular on- and off-target screening of bioactive compounds using microarrays. Org. Biomol. Chem..

[28] Chang M., Wu C., Zheng J., Huang Y. (2016). One-Pot Synthesis of Polysubstituted Benzenes through a *N*,*N*-dimethyl-4-aminopyridine (DMAP)-Catalyzed [4+2] Benzannulation of 1,3-Bis(sulfonyl)butadienes and g-Substituted Allenoates. Chem. Asian J..

[29] Ni H., Yao W., Waheed A., Ullah N., Lu Y. (2016). Enantioselective [4+2]-Annulation of Oxadienes and Allenones Catalyzed by an Amino Acid Derived Phosphine: Synthesis of Functionalized Dihydropyrans. Org. Lett..

[30] Meng X., Jiang S., Xu X.-Y., Wu Q.-X., Gu Y.-C., Shi D.-Q. (2016). DABCO catalyzed unusual formal [4+2] cycloaddition of 3-acyl(or alkoxycarbonyl)-1,4-enediones with 2,3-butadienoates: Effective access to highly functionalized pyrans. RSC Adv..

[31] Xu S., Chen J., Shang J., Qing Z., Zhang J., Tang Y. (2015). Divergent amine-catalyzed [2+2] annulation of allenoates with azodicarboxylates: Facile synthesis of 1,2-diazetidines. Tetrahedron Lett..

[32] Wang D., Lei Y., Wei Y., Shi M. (2014). A Phosphine-Catalyzed Novel Asymmetric [3+2] Cycloaddition of C,N-Cyclic Azomethine Imines with δ-Substituted Allenoates. Chem. Eur. J..

[33] Wang Y., Yu Z.-H., Zheng H.-F., Shi D.-Q. (2012). DABCO and Bu_3_P catalyzed [4+2] and [3+2] cycloadditions of 3-acyl-2Hchromen-ones and ethyl 2,3-butadienoate. Org. Biomol. Chem..

[34] Meng X., Huang Y., Chen R. (2009). Bifunctional Phosphine-Catalyzed Domino Reaction: Highly Stereoselective Synthesis of cis-2,3-Dihydrobenzofurans from Salicyl N-Thiophosphinyl Imines and Allenes. Org. Lett..

[35] Gao Z., Wang C., Yuan C., Zhou L., Xiao Y., Guo H. (2015). Phosphine-catalyzed [4+1] annulation of 2-tosylaminochalcones with allenoates: Synthesis of trans-2,3-disubstitued indolines. Chem. Commun..

[36] Szeto J., Sriramurthy V., Kwon O. (2011). Phosphine-Initiated General Base Catalysis: Facile Access to Benzannulated 1,3-Diheteroatom Five-Membered Rings via Double-Michael Reactions of Allenes. Org. Lett..

[37] Zhu X.-F., Lan J., Kwon O. (2003). An Expedient Phosphine-Catalyzed [4+2] Annulation: Synthesis of Highly Functionalized Tetrahydropyridines. J. Am. Chem. Soc..

[38] Bi Y., Zhang L.-H., Hamaker L.K., Cook J.M. (1994). Enantiospecific Synthesis of (-)-Alstonerine and (+)-Macroline as Well as a Partial Synthesis of (+)-Villalstonine. J. Am. Chem. Soc..

[39] Bi Y., Cook J.M. (1993). General approach for the synthesis of macroline/sarpagine alkaloids. The total synthesis of (+)-macroline. Tetrahedron Lett..

[40] Zhang L.H., Cook J.M. (1990). General approach to the synthesis of macroline-related alkaloids. Stereospecific total synthesis of (−)-alstonerine. J. Am. Chem. Soc..

[41] Tran Y.S., Kwon O. (2005). An Application of the Phosphine-Catalyzed [4+2] Annulation in Indole Alkaloid Synthesis:  Formal Syntheses of (±)-Alstonerine and (±)-Macroline. Org. Lett..

[42] Villa R.A., Xu Q., Kwon O. (2012). Total synthesis of (±)-hirsutine: application of phosphine-catalyzed imine–allene [4 + 2] annulation. Org. Lett..

[43] Wurz R.P., Fu G.C. (2005). Catalytic Asymmetric Synthesis of Piperidine Derivatives through the [4+2] Annulation of Imines with Allenes. J. Am. Chem. Soc..

[44] Xiao H., Chai Z., Wang H.-F., Wang X.-W., Cao D.-D., Liu W., Lu Y.-P., Yang Y.-Q., Zhao G. (2011). Bifunctional N-Acyl-Aminophosphine-Catalyzed Asymmetric [4+2] Cycloadditions of Allenoates and Imines. Chem. Eur. J..

[45] Henry C.E., Xu Q., Fan Y.C., Martin T.J., Belding L., Dudding T., Kwon O. (2014). Hydroxyproline-Derived Pseudoenantiomeric [2.2.1] Bicyclic Phosphines: Asymmetric Synthesis of (+)- and (−)-Pyrrolines. J. Am. Chem. Soc..

[46] Xu Q., Dupper N.J., Smaligo A.J., Fan Y.C. (2018). Cai, L.; Wang, Z.; Langenbacher, A.D.; Chen, J.-N.; Kwon, O. Catalytic Enantioselective Synthesis of Guvacine Derivatives through [4+2] Annulations of Imines with α-Methylallenoates. Org. Lett..

[47] Yu H., Zhang L., Li Z., Liu H., Wang B., Xiao Y., Guo H. (2014). Phosphine-catalyzed [4+2] cycloaddition of sulfamate-derived cyclic imines with allenoates: Synthesis of sulfamate-fused tetrahydropyridines. Tetrahedron.

[48] Chen X.-Y., Ye S. (2012). Phosphane-Catalyzed [4+2] Annulation of Allenoates with Ketimines: Synthesis of Sultam-Fused Tetrahydropyridines. Eur. J. Org. Chem..

[49] Takizawa S., Arteaga F.A., Yoshida Y., Suzuki M., Sasai H. (2014). Enantioselective Organocatalyzed Formal [4+2] Cycloaddition of Ketimines with Allenoates: Easy Access to a Tetrahydropyridine Framework with a Chiral Tetrasubstituted Stereogenic Carbon Center. Asian J. Org. Chem..

[50] Tran Y.S., Kwon O. (2007). Phosphine-Catalyzed [4 + 2] Annulation:  Synthesis of Cyclohexenes. J. Am. Chem. Soc..

[51] Tran Y.S., Martin T.J., Kwon O. (2011). Phosphine-Catalyzed [4+2] Annulations of 2-Alkylallenoates and Olefins: Synthesis of Multisubstituted Cyclohexenes. Chem. Asian J..

[52] Zhong F., Han X., Wang Y., Lu Y. (2012). Highly enantioselective [4+2] annulations catalyzed by amino acid-based phosphines: Synthesis of functionalized cyclohexenes and 3-spirocyclohexene-2-oxindoles. Chem. Sci..

[53] Xiao H., Chai Z., Cao D., Wang H., Chen J., Zhao G. (2012). Highly enantioselective [4 + 2] cycloadditions of allenoates and dual activated olefins catalyzed by N-acyl aminophosphines. Org. Biomol. Chem..

[54] Baskar B., Dakas P.-Y., Kumar K. (2011). Natural Product Biosynthesis Inspired Concise and Stereoselective Synthesis of Benzopyrones and Related Scaffolds. Org. Lett..

[55] Danda A., Kesava-Reddy N., Golz C., Strohmann C., Kumar K. (2016). Asymmetric Roadmap to Diverse Polycyclic Benzopyrans via Phosphine-Catalyzed Enantioselective [4+2]-Annulation Reaction. Org. Lett..

[56] Yang W., Zhang Y., Qiu S., Zhao C., Zhang L., Liu H., Zhou L., Xiao Y., Guo H. (2015). Phosphine-catalyzed [4+2] cycloaddition of unsaturated pyrazolones with allenoates: A concise approach toward spiropyrazolones. RSC Adv..

[57] Liu H., Liu Y., Yuan C., Wang G.-P., Zhu S.-F., Wu Y., Wang B., Sun Z., Xiao Y., Zhou Q.-L. (2016). Enantioselective Synthesis of Spirobarbiturate-Cyclohexenes through Phosphine-Catalyzed Asymmetric [4+2] Annulation of Barbiturate-Derived Alkenes with Allenoates. Org. Lett..

[58] Chen R., Fan X., Xu Z., He Z. (2017). Facile Synthesis of Spirooxindole-Cyclohexenes via Phosphine-Catalyzed [4+2] Annulation of α-Substituted Allenoates. Chin. J. Chem..

[59] Wang T., Ye S. (2010). Diastereoselective Synthesis of 6-Trifluoromethyl-5,6-dihydropyrans via Phosphine-Catalyzed [4+2] Annulation of r-Benzylallenoates with Ketones. Org. Lett..

[60] Jing C., Na R., Wang B., Liu H., Zhang L., Liu J., Wang M., Zhong J., Kwon O., Guo H. (2012). Phosphine-Catalyzed [3+2] and [4+3] Annulation Reactions of C,N-Cyclic Azomethine Imines with Allenoates. Adv. Synth. Catal..

[61] Li Z., Yu H., Feng Y., Hou Z., Zhang L., Yang W., Wu Y., Xiao Y., Guo H. (2015). Phosphine-catalyzed [4 + 3] cycloaddition reaction of aromatic azomethine imines with allenoates. RSC Adv..

[62] Yuan C., Zhou L., Xia M., Sun Z., Wang D., Guo H. (2016). Phosphine-Catalyzed Enantioselective [4 + 3] Annulation of Allenoates with C,N-Cyclic Azomethine Imines: Synthesis of Quinazoline-Based Tricyclic Heterocycles. Org. Lett..

[63] Li E., Huang Y., Liang L., Xie P. (2013). Phosphine-Catalyzed [4+2] Annulation of γ-Substituent Allenoates: Facile Access to FunctionalizedSpirocyclic Skeletons. Org. Lett..

[64] Meng X., Huang Y., Zhao H., Xie P., Ma J., Chen R. (2009). New Chiral Derivatizing Agents: Convenient Determination of Absolute Configurations of Free Amino Acids by ^1^H-NMR. Org. Lett..

[65] Ma R., Xu S., Tang X., Wu G., He Z. (2011). Phosphine-mediated diverse reactivity of g-substituted allenoates with aldehydes: Syntheses of highly functionalized chromans and (*E*,*E*)-1,3-dienes. Tetrahedron.

[66] Xu S., Zhou L., Ma R., Song H., He Z. (2009). Phosphane-Catalyzed [3+2] Annulation of Allenoates with Aldehydes: A Simple and Efficient Synthesis of 2-Alkylidenetetrahydrofurans. Chem. Eur. J..

[67] Zhang X.-C., Cao S.-H., Wei Y., Shi M. (2011). Phosphine-catalyzed highly diastereoselective [3+2] cyclization of isatinderived electron-deficient alkenes with a-allenic esters. Chem. Commun..

[68] Jose A., Lakshmi K.C.S., Suresh E., Nair V. (2013). Phosphine-Mediated Reaction of 3-Alkyl Allenoates and Diaryl 1,2-Diones: Efficient Diastereoselective Synthesis of Fully Substituted Cyclopentenones. Org. Lett..

[69] Gicquel M., Gomez C., Retailleau P., Voituriez A., Marinetti A. (2013). Synthesis of 3,3’-Spirocyclic Oxindoles via Phosphine Catalyzed [4+2] Cyclizations. Org. Lett..

[70] Ni H., Tang X., Zheng W., Yao W., Ullah N., Lu Y. (2017). Enantioselective Phosphine-Catalyzed Formal [4+4] Annulation of α,β-Unsaturated Imines and Allene Ketones: Construction of Eight-Membered Rings. Angew. Chem. Int. Ed..

[71] Zhang Q., Yang L., Tong X. (2010). 2-(Acetoxymethyl)buta-2,3-dienoate, a Versatile 1,4-Biselectrophile forPhosphine-Catalyzed (4 + n) Annulations with 1,n-Bisnucleophiles (n = 1, 2). J. Am. Chem. Soc..

[72] Han X., Yao W., Wang T., Tan Y.R., Yan Z., Kwiatkowski J., Lu Y. (2014). Asymmetric synthesis of spiropyrazolones through phosphine-catalyzed [4+1] annulation. Angew. Chem. Int. Ed..

[73] Ziegler D.T., Riesgo L., Ikeda T., Fujiwara Y., Fu G.C. (2014). Biphenyl-derived phosphepines as chiral nucleophilic catalysts: Enantioselective [4+1] annulations to form functionalized cyclopentenes. Angew. Chem. Int. Ed..

[74] Kramer S., Fu G.C. (2015). Use of a New Spirophosphine To Achieve Catalytic Enantioselective [4+1] Annulations of Amines with Allenes To Generate Dihydropyrroles. J. Am. Chem. Soc..

[75] Jang H., Liu W., Zhang Sean X. a., Liao W. (2016). Phosphine-catalyzed [4+2] annulations of α-aminonitriles with allenoates: Synthesis of functionalized tetrahydropyridines. Chem. Res. Chin. Univ..

[76] Zhang Y., Tong X. (2017). Construction of Complex 1,3-Cyclohexadienes via Phosphine-Catalyzed (4+2) Annulations of delta-Acetoxy Allenoates and Ketones. Org. Lett..

[77] Zhang X.-N., Deng H.-P., Huang L., Wei Y., Shi M. (2012). Phosphine-catalyzed asymmetric [4+1] annulation of Morita-Baylis-Hillman carbonates with dicyano-2-methylenebut-3-enoates. Chem. Commun..

[78] Chen P., Zhang J., Zhang J. (2018). Highly Substituted Cyclohexenes via Phosphine-Catalyzed [4+2] Annulation of Electron-deficient Dienes and Vinyl Ketones. Adv. Synth. Catal..

[79] Li Y., Su X., Zhou W., Li W., Zhang J. (2015). Amino acid derived phosphine-catalyzed enantioselective 1,4-dipolar spiroannulation of cyclobutenones and isatylidenemalononitrile. Chem. Eur. J..

[80] Zhu X.-F., Schaffner A.-P., Li R.C., Kwon O. (2005). Phosphine-Catalyzed Synthesis of 6-Substituted 2-Pyrones: Manifestation of E/Z-Isomerism in the Zwitterionic Intermediate. Org. Lett..

[81] Hu F.-L., Wei Y., Shi M. (2014). Phosphine-Catalyzed Asymmetric Formal [4+2] Tandem Cyclization of Activated Dienes with Isatylidenemalononitriles: Enantioselective Synthesis of Multistereogenic Spirocyclic Oxindoles. Adv. Synth. Catal..

[82] Zhong Y., Zhao X., Gan L., Hong S., Jiang X. (2018). Phosphine-Catalyzed Enantioselective [4+2] Cycloaddition-Semipinacol-Type-Rearrangement Reaction of Morita-Baylis-Hillman Carbonates. Org. Lett..

